# Crystal Structures and Reference Powder Patterns of BaR_2_ZnO_5_ (R = La, Nd, Sm, Eu, Gd, Dy, Ho, Y, Er, and Tm)

**DOI:** 10.6028/jres.104.011

**Published:** 1999-04-01

**Authors:** J. A. Kaduk, W. Wong-Ng, W. Greenwood, J. Dillingham, B. H. Toby

**Affiliations:** Amoco Corporation, Naperville, IL 60566; National Institute of Standards and Technology, Gaithersburg, MD 20899-0001; Geology Department, University of Maryland, College Park, MD 20742; National Institute of Standards and Technology, Gaithersburg, MD 20899-0001

**Keywords:** BaR_2_ZnO_5_(R = lanthanides), crystal structure, x-ray and neutron Rietveld refinements, x-ray reference powder patterns

## Abstract

Reference x-ray powder patterns and the crystal structures of the lanthanide compounds, BaR_2_ZnO_5_, in which R = La, Nd, Sm, Eu, Gd, Dy, Ho, Y, Er, or Tm, were determined by the x-ray Rietveld refinement technique. A structural trend was confirmed for this series of compounds. The compounds with smaller ionic radii (R = Sm, Eu, Gd, Dy, Ho, Y, Er, or Tm) are isostructural to the orthorhombic “green phase” (BaY_2_CuO_5_). The lattice parameters for compounds with R = Tm to Sm range from *a* = 7.01855(9) Å to 7.20452(14) Å, *b* = 12.25445 (17) Å to 12.5882(2) Å, and *c* = 5.6786(14) Å to 5.81218(11) Å, respectively. R is sevenfold coordinated inside a monocapped trigonal prism. These prisms share edges to form wave-like chains parallel to the long *b*-axis. The BaR_2_ZnO_5_ compounds which contain larger size R (La and Nd) crystallize in the tetragonal space group *I4/mcm*. The lattice parameters are *a* = 6.90982(10) and *c* = 11.5977(2) Å for BaLa_2_ZnO_5_, and *a* = 6.75979(5) Å and *c* = 11.54560(12) Å for BaNd_2_ZnO_5_. The structure consists of ZnO_4_ tetrahedra (instead of planar CuO_4_ groups as found in BaR_2_CuO_5_) with 10-fold coordinated bicapped square prismatic Ba and 8-fold coordinated bicapped trigonal prismatic R ions between them. The reference x-ray powder patterns will be submitted to the Powder Diffraction File (PDF).

## 1. Introduction

Extensive structural and property investigations involving substitution of Cu in the Ba_2_RCu_3_O_6+_*_x_* system by various transition metals including Ti, Cr, Mn, Fe, Co, Ni, Au, and Zn have been carried out in order to understand the correlations between superconducting properties and crystal chemistry [1–4]. When the Cu^2+^ (nine 3*d* electrons) of Ba_2_YCu_3_O_6+_*_x_* is completely substituted by Zn^2+^ (ten 3*d* electrons), the sample does not become superconducting, presumably the result of filling of electronic bands. A strong correlation between superconductivity and electronic and magnetic properties of the substituting elements was reported by Xiao et al. [1]. Therefore, studies of the structure and properties of the Zn-analogs should enhance understanding of the factors contributing to superconductivity.

Successful replacement of Cu by Zn in the lanthanide Ba-R-Cu-O system has been described [5–9]. According to Michel et al. [5–8], selected barium lanthanum zinc oxides apparently isostructural to the “green phases”, BaR_2_CuO_5_ [2], can be prepared. The structures of the La, Nd, and Y-compounds have been studied using x-ray powder diffraction [5,6] and neutron powder diffraction methods [9]. It was found that, while the structures of the La and Nd analogs are tetragonal, the Y-compound is orthorhombic. Neutron diffraction studies of selected lanthanide analogs (Dy, Ho, Y, and Er) have also been reported [9]. High neutron absorption cross sections meant that compounds containing the lanthanide ions with larger ionic radius such as Sm, Eu, and Gd were not studied. Michel and Raveau [8] further reported that, while in the Cu-containing system BaR_2_CuO_5_ the orthorhombic structure can be prepared for R = Er, Tm, Yb and Lu, attempts to replace Cu with Zn did not succeed for these compounds. Recently we reported that the Er-analog of BaR_2_ZnO_5_ has been prepared [9].

As the x-ray powder diffraction technique is of primary importance for phase characterization, extensive coverage by accurate reference diffraction patterns of superconductor and related phases in the Powder Diffraction File (PDF) [10] is essential for the high-*T*_c_ superconductivity community. Presently, no reference diffraction pattern other than R = La is available in the PDF for phase identification for BaR_2_ZnO_5_.

The main goals of this investigation were: to supplement the reference diffraction patterns and crystal structures of the BaR_2_ZnO_5_ series by using x-ray Rietveld refinement techniques [11]; to determine the structural details of the analogs with R = Sm, Eu, and Gd; and to investigate the possibility of preparing the analogs of the lanthanide ions with smaller ionic radius (R = Tm, Yb, and Lu).

## 2. Experimental Details

### 2.1 Sample Preparation

Eleven polycrystalline samples of the BaR_2_ZnO_5_ series (R = La, Nd, Sm, Eu, Gd, Dy, Ho, Y, Er, Tm, Yb, and Lu) were prepared by a solid-state sintering method. Well-mixed stoichiometric powders of BaCO_3_, R_2_O_3_, and ZnO were compacted by pressing the powder in a pelletizing die at about 0.3 GPa. The compacted powders were heated in air according to the schedule shown in Table 1. Each time the samples were taken out of the furnace, they were reground and repelletized. About 4 g to 5 g of each of the samples was prepared except for the Er, Tm, Yb, and Lu samples, for which only about a 1 g sample was attempted in order to investigate the feasibility of sample preparation. The colors of these materials are also reported in Table 1. When Cu is replaced by Zn, the color of the phases changes from dark green or brown to the much lighter colors of cream, blue, beige, or peach.

### 2.2 X-Ray Powder Studies

**Fig. 1:**
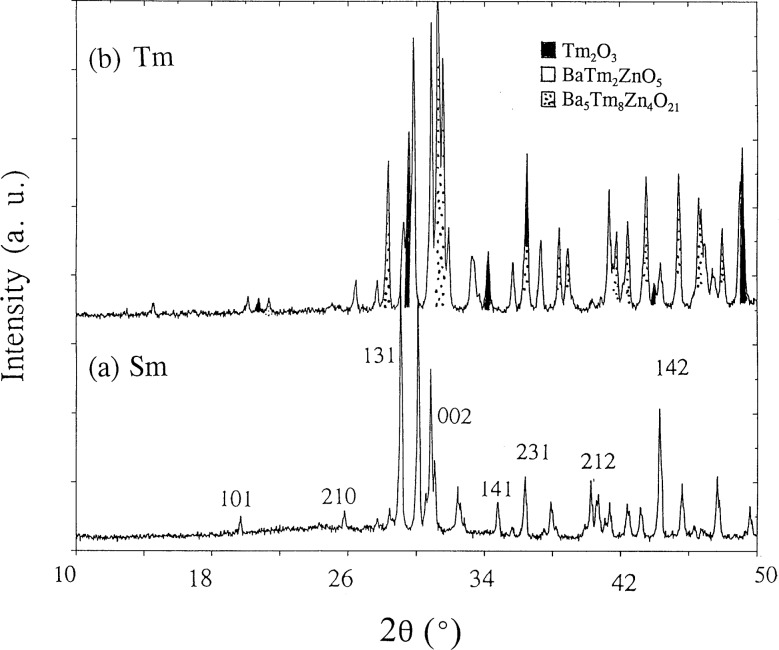
X-ray diffraction patterns of (a) BaTm_2_ZnO_5_ and (b) BaSm_2_ZnO_5_. The impurity phases are marked.

X-ray powder diffraction was used to identify the phases synthesized and to confirm phase purity. The PDF reference x-ray diffraction pattern of BaY_2_ZnO_5_ was used for performing phase identification. While the La, Nd, and Y preparations were phase-pure, small concentrations of binary oxides were observed in the Eu, Dy, Ho, and Er products. A minor concentration of an unidentified phase was detected in the Sm, Eu, and Gd products. The Tm preparation contained significant concentrations of Ba_5_Zn_4_Tm_8_O_21_ [13,14] and Tm_2_O_3_ (see Fig. 1a). The Yb and Lu preparations yielded only Ba_5_Zn_4_R_8_O_21_ and R_2_O_3_.

For the Rietveld refinements, the powders were mounted in zero-background quartz holders with double-sided adhesive tape. A Scintag PAD V diffractometer^1^ equipped with an Ortec intrinsic Ge detector was used to measure the powder patterns (Cu K_α_ radiation, 40 kV, 30 mA) for values of 2*θ* from 3°–140° in 0.02° steps, counting after each step for 10 s or 12 s.

All data processing was carried out using the GSAS software suite [12]. To minimize the effects of surface roughness and incomplete interception of the beam, only the 18° to 140° portions of the patterns were used in the refinements. The initial structure models were taken from Ref. [9]. For the La and Nd compounds, the tetragonal space group *I4/mcm* was used, while for compounds of the smaller lanthanides, the orthorhombic space group *Pbnm* (an alternate setting of *Pnma*) was used.

All atomic positions were refined isotropically. The displacement coefficients of the two independent lanthanide ions in *Pbnm* were constrained to have a common value. In all refinements, a single isotropic displacement coefficient was refined for the oxygen atoms. A scale factor and the lattice parameters were refined for the major phase. In some samples, impurity phases were detected, and were subsequently included in the refinements using fixed structural models. The peak profiles were described using a pseudo-Voigt function. Only the Cauchy *X*, asymmetry, and sample displacement parameters were refined. Backgrounds were described using a 3-term cosine Fourier series.

## 3. Results and Discussion

**Fig. 2:**
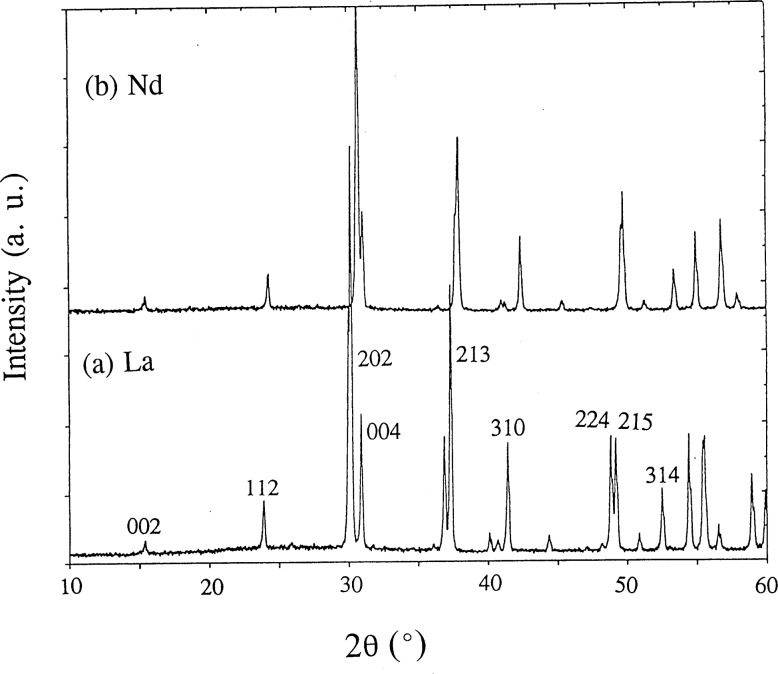
X-ray diffraction patterns of (a) BaLa_2_ZnO_5_ and (b) BaNd_2_ZnO_5_.

As the ionic size of R in BaR_2_ZnO_5_ decreases, it becomes progressively more difficult to prepare the BaR_2_ZnO_5_ phase. This phase cannot be prepared for R = Yb and Lu under the specified conditions. We believe that under more appropriate heat treatment conditions (i.e., different oxygen partial pressure), a single phase can be formed in the Ba-Tm-Zn system. This possibility is under investigation. Since the refined structural parameters for BaTm_2_ZnO_5_ are not as accurate or precise as those derived from the pattern of a pure phase, these parameters will not be discussed. The x-ray diffraction pattern of the nominal single phase BaSm_2_ZnO_5_ is illustrated in Fig. 1b; selected Miller indices are indicated. Figures 2a and 2b show the x-ray diffraction patterns of BaLa_2_ZnO_5_ and BaNd_2_ZnO_5_. The patterns of these analogs are similar. The small displacement of the corresponding peaks in the La and Nd patterns indicates the effect of ionic size on the isostructural compounds.

**Fig. 3:**
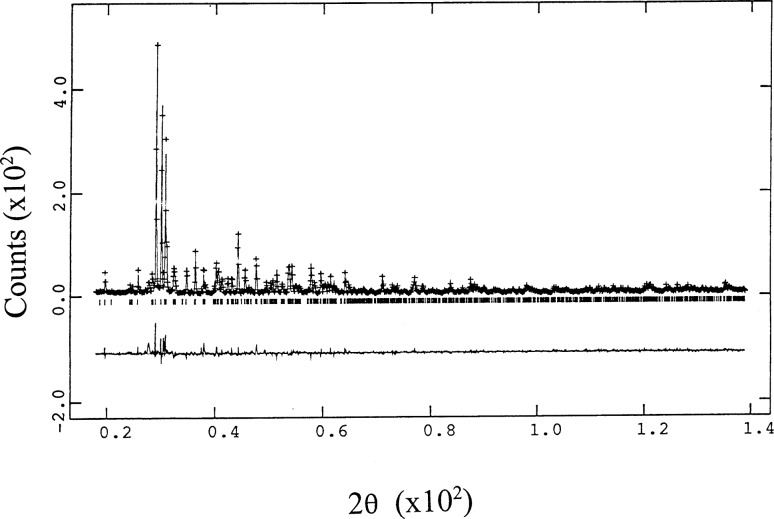
Rietveld refinement results for BaSm_2_ZnO_5_. The upper graph shows the fit between the experimental and calculated patterns while the lower graph shows the difference between these two patterns.

The refinement residuals using the GSAS suite [12] are reported in Table 2. The observed, calculated, and difference pattern of BaSm_2_ZnO_5_ is illustrated in Fig. 3. The upper graph shows the fit between the experimental and calculated patterns while the lower one shows the difference between these two patterns. The refined structural parameters for the tetragonal compounds are reported in Table 3, and those of the orthorhombic phases are reported in Table 4. Global parameters are given in Table 5. Selected structural quantities such as bond lengths and bond angles are reported in Tables 6 and 7. Also reported in the above tables are results of the neutron refinements of the lattice parameters [9] and bond lengths and angles for the R = La, Nd, Dy, Ho, Y, and Er compounds for comparison wherever appropriate. Although the refined overall structure, structural parameters, and bond distances derived from the x-ray and neutron refinements [9] for the tetragonal structures agree quite well, there are differences in the bond distances for some of the orthorhombic structures. These small differences are most likely associated with the relatively large uncertainties in the determination of the oxygen positions by using x-ray techniques.

**Figure 4:**
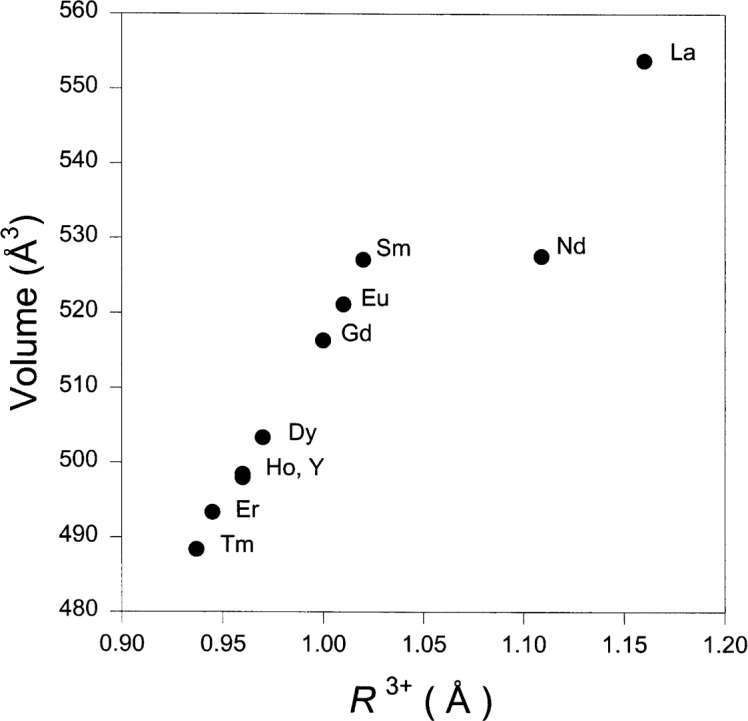
Plot of unit cell volume vs *R*^3+^ (R = La, Nd, Sm, Eu, Gd, Dy, Y, Ho, Er and Tm) as found in BaR_2_ZnO_5_. *R*^3+^ is the ionic radius for coordination number (C.N.) = 8 for the La and Nd compounds and C.N. = 7 for the rest [15,16].

The trend of unit cell volume with the ionic radius, *R*^3+^ of BaR_2_ZnO_5_ (where *R*^3+^ = La, Nd, Sm, Eu, Gd, Dy, Ho, Y, Er, and Tm) is illustrated in Fig. 4. These ionic radii are chosen based on the structural environment surrounding these ions, namely, 8-fold coordination for R = La and Nd, and 7-fold coordination for the rest of the smaller ions [15,16]. Since the 7-fold coordination radii for the Ho and Tm analogs were not reported, they were estimated by interpolating between those for 6- and 8-fold coordination. A monotonic decrease of the volumes vs the ionic radius from Sm to Tm is observed, agreeing well with the trend expected from lanthanide contraction. Since the La and Nd analogs adopt a different structure, the cell parameters of these two compounds do not fall on the same line as the smaller analogs.

### 3.1 Crystal Structures of BaR_2_ZnO_5_

It has been reported that, since the effective ionic radii of Zn^2+^ (0.68 Å for coordination number (C.N.) of 5, and 0.60 Å for C.N. = 4) and Cu^2+^ (0.65 Å for C.N. = 5, and 0.57 Å for C.N. = 4) are comparable [15,16], it is possible for the Zn series and the Cu series (BaR_2_CuO_5_) to be isostructural. Results of both neutron and x-ray Rietveld refinements showed that the BaR_2_ZnO_5_ compounds with smaller R are indeed isostructural to the “green phase” compounds [6,8,9,17]. When R = La and Nd, the structures of BaR_2_ZnO_5_ are different from the “brown phase” BaR_2_CuO_5_ analogs. The refined atomic coordinates for the La and Nd compounds agree well with those reported by Michel et al. using x-ray diffraction [6,8] and Wong-Ng et al. [9].

#### 3.1.1 Structure of Tetragonal BaR_2_ZnO_5_, R = La or Nd

**Figure 5:**
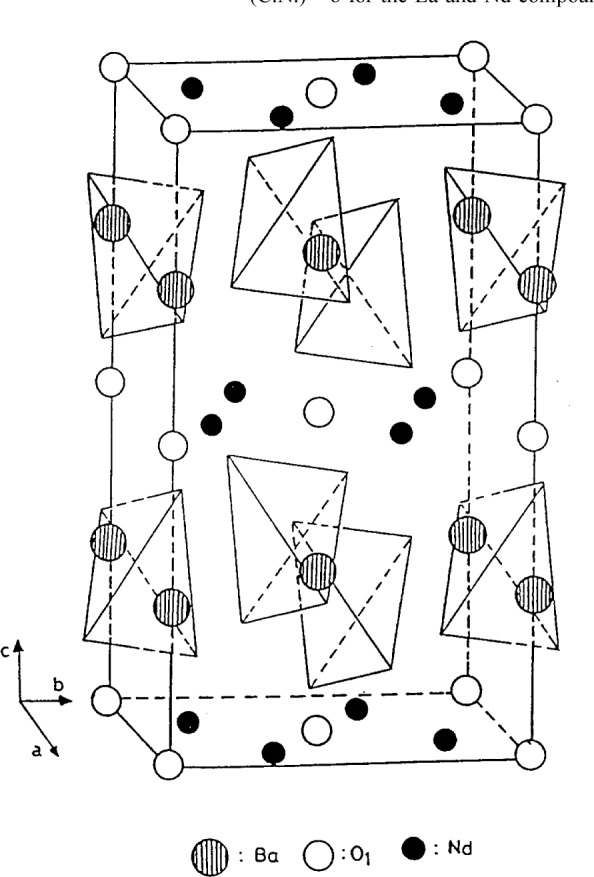
Structure of BaNd_2_ZnO_5_, showing the ZnO_4_ tetrahedra. Ba ions are located between these tetrahedra.

Detailed descriptions of the structures of BaLa_2_ZnO_5_ and BaNd_2_ZnO_5_ have been reported by Michel et al. [6,7], Taibi et al. [18], and Wong-Ng et al. [9] (Fig. 5). Despite similar effective ionic sizes of Zn^2+^ and Cu^2+^, the structures of BaR_2_ZnO_5_ and the brown phase BaR_2_CuO_5_ compounds are different [5,19]. The brown phase crystallizes in the space group *P4/mbm*, and the structure contains square planar CuO_4_ groups. The Zn analogs consist of a three-dimensional array of interconnected BaO_10_ and RO_8_ polyhedra as well as tetrahedral ZnO_4_ groups (see Fig. 5). The RO_8_ polyhedron is a trigonal prism capped on two of the three rectangular faces, and the capped BaO_10_ polyhedron is a square prism capped on both ends by tetragonal pyramids. The structure can be viewed as consisting of alternate layers of Zn-Ba-O and R-O extending infinitely in the *ab* plane and perpendicular to the *c* axis.

The atomic valence values *V_b_* for Ba, R/R1, R2, and Zn were calculated [20] and are listed in Table 8. The *V_b_* of an atom *i* is defined as the sum of the bond valences *v_ij_* of all the bonds from atom *i* to atoms *j* (*V_bi_* = Σ *v_ij_*). The most commonly adopted empirical expression for the bond valence *v_ij_* as a function of the interatomic distance *d_ij_* is

$$v_{ij}=\exp\left[\left(r_{0}-d_{ij}\right)/B\right].$$

The parameter *B* is commonly taken to be a “universal” constant equal to 0.37 Å [20,21]. The values of the reference distance *r*_0_ are tabulated for various pairs of atoms [20]. The *V_b_* values (1.686 and 1.784) of Ba in the La and Nd analogs indicate an underbonded situation in both compounds. These values, which agree with those derived from neutron data, are significantly less than the expected value of 2 and indicate the size of the BaO_10_ cages are relatively large. The *V_b_* values for Zn (2.108 and 2.102 in the La- and Nd-analogs) are very close to the expected value of 2.

#### 3.1.2 Structure of Orthorhombic BaR_2_ZnO_5_, R = Sm, Eu, Gd, Dy, Ho, Y, or Er

The structures of the orthorhombic BaR_2_ZnO_5_ compounds are similar to those of the “green phase” BaR_2_CuO_5_ analogs. The detailed structure of the green phase type structure has been reported [6,7,9,22,23,24]. The basic structure of these compounds consists of RO_7_, BaO_11_, and ZnO_5_ polyhedra. R is 7-fold coordinated inside a monocapped trigonal prism, and two such units join to form the basic structure motif of R_2_O_11_. The Ba atoms are found to reside in distorted 11-fold coordinated cages, characterized by Ba–O distances between 2.650(20) Å and 3.346(17) Å, where the values inside the brackets are standard uncertainties. Both CuO_5_ and ZnO_5_ in BaR_2_CuO_5_ and BaR_2_ZnO_5_ have a distorted tetragonal pyramidal coordination. Similar to the La-analog, the *V*_b_ values for Ba are less than 2 (Table 8). The Zn–O distances in the seven compounds range from 1.950(11) Å to 2.164(13) Å. The *V*_b_ values for R and Zn do not deviate significantly from the expected values of 3 and 2, respectively, except for the Eu-analog. In this compound, the Eu2-O polyhedron appears to be relatively small (overbonded, with *V_b_* = 3.353), whereas the size of the Zn-O square pyramid is relatively large (*V_b_* = 1.687). This result may be due to the relatively large uncertainty associated with the position of O3 derived from the x-ray data.

**Figure 6:**
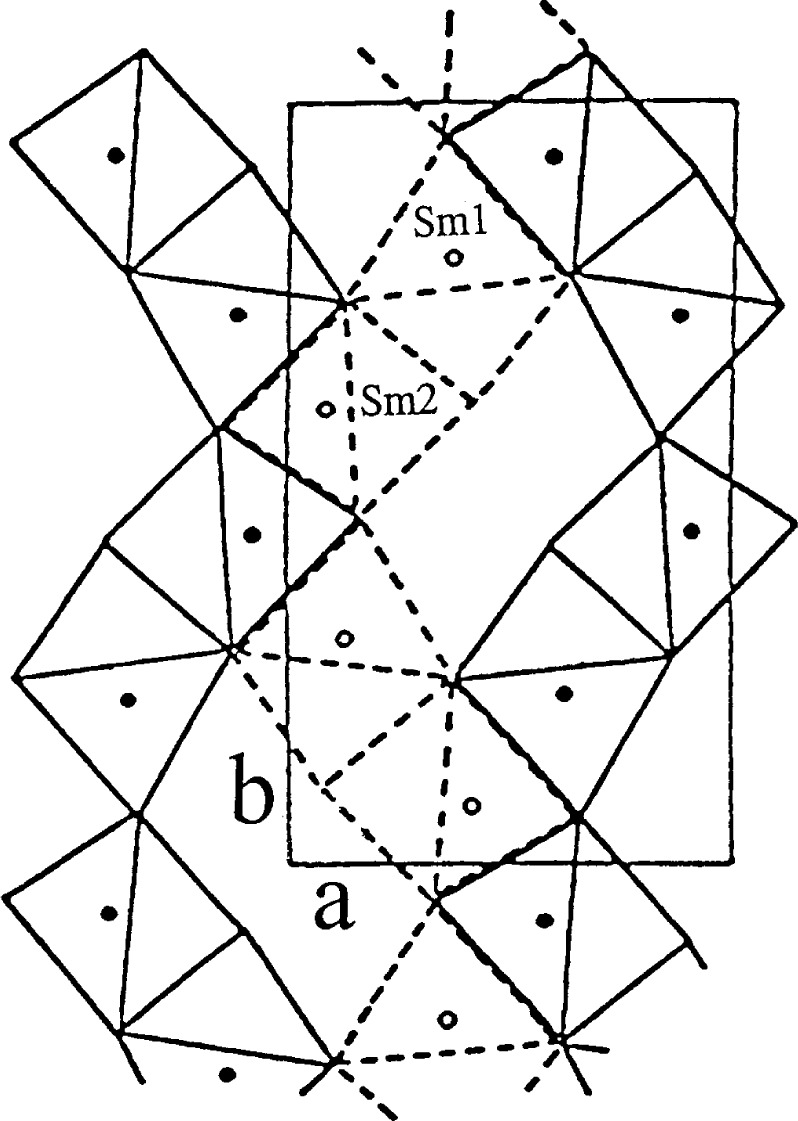
Projection of the structure of BaSm_2_ZnO_5_ in the (001) plane showing the linkage of R_2_O_11_ polyhedra at *z* = 1/4 (broken line) and *z* = 3/4 (solid line). The wave-like chains along the *b*-axis are displayed, and the two independent Sm atoms are labelled.

In Fig. 6, the projection of the Sm_2_O_11_ blocks at *z* = 1/4 is shown as solid lines and the second layer at *z* = 3/4 is represented as dotted lines. These prisms share edges to form wave-like chains parallel to the long *b*-axis. Chains are crosslinked by Cu and Ba atoms. The *c* direction is the shortest axis, and is also the direction in which layers of prisms are stacked parallel to each other, sharing the trigonal faces.

### 3.2 Reference X-Ray Diffraction Patterns

Reference x-ray powder patterns of the nine compounds BaR_2_ZnO_5_, in which R = La, Nd, Sm, Eu, Gd, Dy, Ho, Y, or Er, were obtained using a pattern decomposition technique. Because the refined structural parameters for the R = Tm analog are not as accurate or precise as those derived from the pattern of a pure or nearly pure phase, the x-ray diffraction pattern of this phase is not reported here. These patterns represent ideal specimen patterns. They are corrected for systematic errors both in *d* and *I*. The reported peak positions are calculated from the refined lattice parameters, as this represents the best measure of the true positions. For peaks resolved at the instrument resolution function, the individual peak positions are reported. For overlapping peaks, the intensity weighted average peak position is reported with multiple indices. For marginally resolved peaks, individual peaks are reported, to more accurately simulate the visual appearance of the pattern.

Tables 9 to 17 list these patterns with *d* spacings, Miller indices *h*, *k*, *l* and integrated intensities *I*, normalized to the value 999 as the maximum. The symbols M and + refer to peaks containing contributions from two and more than two reflections, respectively. These patterns have been submitted to International Centre for Diffraction Data (ICDD) for inclusion in the PDF.

## 4. Summary

The reference x-ray diffraction patterns and the crystal structures of both tetragonal and orthorhombic BaR_2_ZnO_5_, R = La, Nd, Sm, Eu, Gd, Dy, Ho, Y, or Er were obtained by Rietveld refinement. The most striking difference between the orthorhombic and tetragonal BaR_2_ZnO_5_ structures is the Zn-O coordination environment. In the orthorhombic structure, the Zn atom is coordinated to five oxygen atoms, four of which form the base of a square pyramid, whereas in the tetragonal structure, the Zn atom is tetrahedrally coordinated.

## Figures and Tables

**Table 1:** Heat treatment scheme for BaR_2_ZnO_5_ (R = La, Nd, Sm, Eu, Gd, Dy, Ho, Y, Er, or Tm). The duration of annealing (number of days) is given in parenthesis

R	Color			Temp. (8C)			
La	white	850(2)	950(3)	1000(1.5)			
Nd	pale blue	850(2)	950(3)	1000(1.5)			
Sm	cream	850(2)	950(3)	1000(1.5)			
Eu	grey	850(2)	950(3)	1000(1.5)			
Gd	beige	850(2)	950(3)	1000(1.5)			
Dy	grey	850(2)	950(3)	1000(1.5)	1080(5)	1090(1.5)	
Ho	pink	850(2)	950(3)	1000(1.5)	1080(5)	1090(1.5)	
Y	beige	850(2)	950(3)	1000(1.5)	1080(5)	1090(1.5)	1100(7)
Er	lavender	850(2)	950(3)	1000(1.5)	1080(5)	1090(1.5)	
Tm	cream	850(2)	950(3)	1000(1.5)		1100(1.5)	1200(3)
Yb	cream	850(2)	950(3)	1000(1.5)		1100(1.5)	1200(3)
Lu	cream	850(2)	950(3)	1000(1.5)		1100(1.5)	1200(3)

**Table 2:** Rietveld refinement residuals for BaR_2_ZnO_5_ using the GSAS software system. The ionic radius of R (*R*^+3^) used are 8-fold coordinated for R = La or Nd and 7-fold coordinated for the rest of the smaller ions [16]. The values for the Ho and Tm analogs were estimated by using the interpolated values between those of the 6- and 8-fold coordination

R	La	Nd	Sm	Eu	Gd	Dy	Ho	Y	Er
R^3+^(Å)	1.160	1.109	1.02	1.01	1.00	0.97	0.96	0.96	0.945
wRp	0.0997	0.1339	0.1486	0.1184	0.0811	0.0733	0.0734	0.1145	0.0712
Rp	0.0749	0.0899	0.1107	0.0893	0.0627	0.0577	0.0568	0.0853	0.0524
R(F)	0.0593	0.0436	0.0592	0.0536	0.0374	0.0493	0.0353	0.0408	0.0369
*χ*^2^	1.756	2.355	2.912	1.927	1.431	1.703	1.883	3.918	2.463
No. var.	15	15	30	35	30	33	32	30	33
D*F*+/−	4.4/−3.6	4.1/−2.2	3.6/−2.7	4.9/−2.8	3.0/−2.0	2.5/−2.5	4.4/−1.8	1.6/−2.2	1.8/−1.9

**Table 3:** Refined Structural Parameters of tetragonal BaR_2_ZnO_5_ (Space Group *I4/mcm*). Values in italics are from the neutron refinements. The numbers inside the parenthesis are standard uncertainties. *Z* is the number of formulas per unit cell. Uiso is the isotropic temperature factor

R	La	Nd
Cell parameter, *a* (Å)	6.90991(7)	6.75982(4)
	*6.9118(1)*	*6.7608(1)*
*c* (Å)	11.59782(16)	11.54565(11)
	*11.6002(2)*	*11.5442(2)*
*V* (Å^3^)	553.759	527.580
Ba, 00¼, Uiso (Å^2^)	0.0048(4)	0.0042(4)
	*0.0098(5)*	*0.0105(5)*
R, *x*(½ + *x*)0, *x*	0.1737(1)	0.1743(1)
	*0.1743(1)*	*0.1744(1)*
Uiso (Å^2^)	0.0030(3)	0.0024(2)
	*0.0073(2)*	*0.0066(3)*
Zn, 0½¼, Uiso (Å^2^)	0.0115(9)	0.0056(7)
	*0.0049(4)*	*0.0091(5)*
O1, 000, Uiso (Å^2^)	0.0039(18)	0.0044(16)
	*0.0106(5)*	*0.0121(6)*
O2, *x*(½+ *x*)*z*, *x*	0.3548(9)	0.3564(8)
	*0.3553(1)*	*0.3547(1)*
*z*	0.1357(6)	0.1310(6)
	*0.1337(1)*	*0.1314(1)*
Uiso (Å^2^)	0.0039(18)	0.0044(16)
	*0.0115(3)*	*0.0118(3)*

**Table 4:** Refined structural parameters of the orthorhombic BaR_2_ZnO_5_ compounds (space group *Pbnm*). Values in italics are from neutron/synchrotron (Er-analog) refinements. The numbers inside the parenthesis are standard uncertainties. *Z* is the number of formulas per unit cell. Uiso is the isotropic temperature factor

R	Sm	Eu	Gd	Dy	Ho	Y	Er	Tm
Cell, *a* (Å)	7.20447(14)	7.17890(10)	7.15729(9)	7.09330(7)	7.07113(6)	7.07018(7)	7.04515(7)	7.01855(9)
				* 7.0944(1)*	* 7.0713(1)*	* 7.0707(1)*	* 7.0472(1)*	
*b* (Å)	12.58817(23)	12.53575(17)	12.49393(15)	12.38464(13)	12.34199(11)	12.33678(12)	12.29815(12)	12.25445(17)
				*12.3885(2)*	*12.3437(2)*	*12.3386(1)*	*12.3022(1)*	
*c* (Å)	5.81214(10)	5.79103(8)	5.77424(7)	5.72979(6)	5.71158(5)	5.70908(5)	5.69410(5)	5.67786(14)
				*5.7314(1)*	*5.7120(2)*	*5.7095(1)*	*5.6958(1)*	
*V* (Å^3^	527.109	521.152	516.348	503.350	498.460	497.964	493.350	488.344
Ba, *xy*¼, *x*	0.9246(3)	0.9238(3)	0.9241(2)	0.9240(3)	0.9238(2)	0.9238(2)	0.9239(2)	0.9235(4)
				*0.9252(10)*	*0.9228(5)*	*0.9231(4)*	*0.9229(2)*	
*y*	0.9012(2)	0.9009(2)	0.9006(1)	0.8999(2)	0.8994(1)	0.8998(1)	0.8989(1)	0.8999(2)
				*0.9007(5)*	*0.9001(3)*	*0.9001(2)*	*0.8995(1)*	
Uiso, Å^2^	0.0071(7)	0.0059(6)	0.0062(5)	0.0039(6)	−0.0001(4)	0.0039(4)	0.0049(5)	0.0015(20)
				*0.020(2)*	*0.0103(9)*	*0.0093(6)*	*0.0059(3)*	
R1, *xy*¼, *x*	0.1186(3)	0.1182(3)	0.1196(3)	0.1206(3)	0.1202(2)	0.1204(3)	0.1205(2)	0.1208(5)
				*0.1210(4)*	*0.1206(3)*	*0.1201(2)*	*0.1209(2)*	
*y*	0.2920(2)	0.2925(2)	0.2930(1)	0.2926(2)	0.2924(1)	0.2919(2)	0.2924(1)	0.2935(2)
				*0.2927(2)*	*0.2918(2)*	*0.2919(1)*	*0.2923(1)*	
Uiso, Å^2^	0.0041(4)	0.0030(3)	0.0017(3)	0.0020(3)	0.0096(3)	0.0018(3)	0.0022(3)	0.002
				*0.0170(6)*	*0.0047(5)*	*0.0055(3)*	*0.0033(2)*	
R2, *xy*¼, *x*	0.3965(3)	0.3977(3)	0.3977(2)	0.3992(3)	0.3997(2)	0.3988(3)	0.4001(2)	0.3963(4)
				*0.3984(3)*	*0.3988(3)*	*0.3989(2)*	*0.3994(1)*	
*y*	0.0750(2)	0.0747(2)	0.0745(1)	0.0740(2)	0.0746(1)	0.0735(2)	0.0744(1)	0.0722(2)
				*0.0744(2)*	*0.0741(2)*	*0.0739(1)*	*0.0743(1)*	
Uiso, Å^2^	0.0041(4)	0.0030(3)	0.0017(3)	0.0020(3)	0.0096(3)	0.0018(3)	0.0022(3)	0.002
				*0.0157(5)*	*0.0043(5)*	*0.0063(3)*	*0.0028(2)*	
Zn, *xy*¼, *x*	0.6890(7)	0.6908(6)	0.6909(5)	0.6920(6)	0.6936(4)	0.6910(4)	0.6927(5)	0.6908(9)
				*0.6984(9)*	*0.6904(4)*	*0.6907(3)*	*0.6902(3)*	
*y*	0.6499(4)	0.6497(4)	0.6490(3)	0.6502(3)	0.6501(2)	0.6499(2)	0.6493(3)	0.6555(6)
				*0.6492(6)*	*0.6505(3)*	*0.6501(2)*	*0.6501(2)*	
Uiso, Å^2^	0.0071(16)	0.0076(14)	0.0046(12)	0.0059(12)	−0.0022(8)	0.0073(8)	0.0041(10)	0.0011(20)
				*0.0179(13)*	*0.0074(6)*	*0.0061(4)*	*0.0035(5)*	
O1, *x*	0.1692(22)	0.1662(18)	0.1657(15)	0.1754(16)	0.1645(12)	0.1578(12)	0.1744(14)	0.1720(26)
				*0.1678(6)*	*0.1669(3)*	*0.1663(2)*	*0.1666(13)*	
*y*	0.4292(17)	0.4368(14)	0.4323(11)	0.4333(13)	0.4315(9)	0.4323(8)	0.4312(11)	0.4231(25)
				*0.4343(5)*	*0.4347(2)*	*0.4344(1)*	*0.4323(11)*	
*z*	−0.0226(29)	−0.0052(23)	−0.0018(19)	−0.0083(21)	−0.0085(16)	−0.0036(16)	−0.0050(18)	−0.026(4)
				−*0.0008(8)*	−*0.0036(4)*	−*0.0024(3)*	*0.000(2)*	
Uiso, Å^2^	0.014(3)	0.006(2)	0.004(2)	0.005(2)	0.01	0.0031(14)	0.003(2)	0.012(4)
				*0.019(1)*	*0.0093(4)*	*0.0074(3)*	*0.010(2)*	
O2, *x*	0.3566(26)	0.3530(21)	0.3547(18)	0.3554(20)	0.3574(15)	0.3503(13)	0.3607(17)	0.367(4)
				*0.3586(8)*	*0.3592(3)*	*0.3586(2)*	*0.3579(15)*	
*y*	0.2174(15)	0.2216(12)	0.2225(10)	0.2261(11)	0.2261(8)	0.2281(7)	0.2250(9)	0.2310(19)
				*0.2249(4)*	*0.2249(2)*	*0.2251(1)*	*0.2240(8)*	
*z*	0.5020(31)	0.5025(25)	0.5023(21)	0.4899(22)	0.5015(17)	0.5054(18)	0.5030(19)	0.499(5)
				*0.5058(10)*	*0.5020(5)*	*0.5014(3)*	*0.5045(2)*	
Uiso, Å^2^	0.014(3)	0.006(2)	0.004(2)	0.005(2)	0.01	0.0031(14)	0.003(2)	0.012(4)
				*0.022(1)*	*0.0097(5)*	*0.0093(3)*	*0.006(2)*	
O3, *xy*¼, *x*	0.0663(34)	0.0833(29)	0.0748(24)	0.0755(26)	0.0690(19)	0.0817(20)	0.0776(22)	0.110(5)
				*0.0762(11)*	*0.0763(5)*	*0.0752(3)*	*0.0756(2)*	
*y*	0.0996(21)	0.0978(17)	0.0991(14)	0.0954(16)	0.1004(12)	0.0986(11)	0.0997(13)	0.0971(25)
				*0.0999(6)*	*0.1005(3)*	*0.1008(2)*	*0.1024(11)*	
Uiso, Å^2^	0.014(3)	0.006(2)	0.004(2)	0.005(2)	0.01	0.0031(14)	0.003(2)	0.012(4)
				*0.026(2)*	*0.0100(8)*	*0.0077(5)*	*0.006(3)*	

**Table 5:** Refined global parameters of BaR_2_ZnO_5_ using the GSAS software suite [12]. The meaning of the variables listed in this table are given in Ref. [12]. The numbers inside the parenthesis are standard uncertainties. The impurity phases are expressed in mass fraction %.

R	La	Nd	Sm	Eu	Gd	Dy	Ho	Y	Er	Tm
Second phases				3 % Eu_2_O_3_		3 % Dy_2_O_3_	2 % Ho_2_O_3_		5 % Er_2_O_3_	11 %
				0.5 % ZnO						Tm_2_O_3_
			unident.	unident.	unident.					43 %
										Ba_5_Zn_4_Tm_8_O_21_
Profile *X*			3.63(8)	1.60(5)	2.78(5)	0.60(5)	0.88(4)	0.49(4)	0.98(4)	0.26(12)
*Y*	12.7(1)	8.2(1)								
asym			3.6(2)	1.9(2)	3.51(1)	3.5(1)	3.2(1)	2.3(1)	3.5(1)	2.1(1)
BK1	10.05(3)	5.40(3)	6.20(4)	7.48(3)	13.90(4)	22.72(5)	21.20(5)	11.27(6)	31.25(7)	24.79(8)
BK2	1.39(4)	0.62(4)	0.58(5)	0.06(4)	0.02(5)	0.19(7)	0.24(7)	0.21(7)	0.50(9)	−2.28(11)
BK3	4.76(4)	2.38(4)	2.20(4)	1.88(4)	1.96(4)	2.46(6)	1.75(6)	1.79(6)	2.29(8)	1.67(10)

**Table 6:** Selected bond distances (Å) in tetragonal BaR_2_ZnO_5_ compounds. Distances in italics are from the neutron refinements

R	La	Nd
Ba-O1 × 2	2.89945(4)	2.88641(3)
	*2.90004(4)*	*2.88605(4)*
Ba-O2 × 8	2.962(4)	2.939(4)
	*2.9749(6)*	*2.9308(7)*
R-O1 × 2	2.5542(4)	2.4970(3)
	*2.5532(3)*	*2.4971(3)*
R-O2 × 2	2.369(9)	2.306(8)
	*2.3531(14)*	*2.2964(15)*
R-O2 × 4	2.715(6)	2.636(6)
	*2.7038(11)*	*2.6483(12)*
Zn-O2 × 4	1.941(9)	1.942(8)
	*1.9541(11)*	*1.9501(12)*

**Table 7:** Selected bond distances (Å) in Orthorhombic BaR_2_ZnO_5_ compounds. Values in italics are from the neutron/synchrotron (Er-analog) refinements

R	Sm	Eu	Gd	Dy	Ho	Y	Er
Ba-O1 × 2	3.346(17)	3.324(14)	3.300(11)	3.231(12)	3.288(9)	3.318(9)	3.205(10)
				*3.252(8)*	*3.271(4)*	*3.269(3)*	*3.249(10)*
Ba-O1′ × 2	3.113(17)	3.093(14)	3.136(11)	3.049(13)	3.101(9)	3.132(9)	3.067(11)
				*3.091(9)*	*3.069(4)*	*3.076(3)*	*3.094(11)*
Ba-O2 × 2	3.159(19)	3.124(15)	3.094(12)	2.995(13)	3.005(10)	3.027(9)	2.992(11)
				*3.041(8)*	*3.271(4)*	*3.269(3)*	*3.286(11)*
Ba-O2′ × 2	2.900(18)	2.891(15)	2.897(12)	2.929(14)	2.893(10)	2.862(10)	2.885(11)
				*2.904(9)*	*3.020(4)*	*3.018(2)*	*3.025(10)*
Ba-O3	2.699(28)	2.722(22)	2.705(19)	2.650(20)	2.685(15)	2.695(14)	2.696(17)
				*2.689(10)*	*2.702(4)*	*2.699(3)*	*2.718(14)*
Ba-O3′ × 2	2.9068(6)	2.8960(4)	2.88713(6)	2.8655(4)	2.8562(2)	2.8549(2)	2.8471(1)
				*2.866(7)*	*2.856(5)*	*2.855(4)*	*2.848(13)*
R1-O1 × 2	2.372(19)	2.361(15)	2.293(13)	2.319(14)	2.285(10)	2.273(9)	2.273(11)
				*2.292(6)*	*2.271(3)*	*2.297(2)*	*2.258(11)*
R1-O2 × 2	2.443(18)	2.402(15)	2.394(13)	2.411(14)	2.355(10)	2.273(9)	2.372(11)
				*2.387(6)*	*2.364(7)*	*2.363(2)*	*2.365(10)*
R1-O2′ × 2	2.378(19)	2.389(15)	2.382(13)	2.311(14)	2.350(10)	2.321(10)	2.318(12)
				*2.339(6)*	*2.370(3)*	*2.341(2)*	*2.331(10)*
R1-O3	2.450(27)	2.453(21)	2.443(18)	2.463(20)	2.397(15)	2.400(13)	2.388(16)
				*2.409(8)*	*2.304(4)*	*2.379(2)*	*2.358(14)*
R2-O1 × 2	2.471(21)	2.398(13)	2.396(11)	2.401(12)	2.347(11)	2.311(9)	2.384(10)
				*2.302(7)*	*2.306(3)*	*2.291(2)*	*2.301(12)*
R2-O1 × 2	2.369(16)	2.320(16)	2.340(14)	2.347(15)	2.327(9)	2.300(10)	2.342(12)
				*2.388(5)*	*2.367(3)*	*2.263(2)*	*2.363(10)*
R2-O2 × 2	2.333(19)	2.373(15)	2.374(13)	2.352(14)	2.377(10)	2.425(10)	2.363(11)
				*2.389(6)*	*2.338(3)*	*2.371(2)*	*2.362(10)*
R2-O3	2.399(25)	2.275(21)	2.331(18)	2.312(18)	2.360(14)	2.263(14)	2.294(15)
				*2.308(8)*	*2.381(4)*	*2.313(3)*	*2.308(14)*
Zn-O1 × 2	1.944(19)	2.059(15)	2.035(13)	1.967(14)	1.980(10)	2.037(10)	1.950(11)
				*2.034(7)*	*2.026(3)*	*2.027(2)*	*2.019(11)*
Zn-O2 × 2	2.230(19)	2.181(15)	2.174(13)	2.164(13)	2.116(10)	2.074(10)	2.124(11)
				*2.123(8)*	*2.120(4)*	*2.124(2)*	*2.114(10)*
Zn-O3	1.945(24)	2.072(20)	2.002(17)	2.015(17)	1.956(13)	2.029(13)	1.999(15)
				*1.981(10)*	*1.972(10)*	*1.976(3)*	*1.963(14)*

**Table 8:** Atomic valence values (*V_b_*) for the Ba, Zn, and R calculated from the measured bond distances, Å [20, 21]. R1 and R2 are used for the two independent lanthanide sites in the BaR_2_ZnO_5_ compounds containing Sm, Eu, Gd, Dy, Ho, Y, or Er. The values in *italics* refer to calculations based on data from neutron/synchrotron (Er-analog) refinements [9]

R	Ba	R or R1	R2	Zn
La	1.686	3.269		2.108
	*1.641*	*2.890*		*2.035*
Nd	1.784	2.900		2.102
	*1.815*	*2.899*		*2.057*
Sm	1.616	3.005	3.109	2.049
Eu	1.654	2.974	3.352	1.687
Gd	1.673	3.111	3.124	1.826
Dy	1.868	2.923	2.934	1.991
	*1.783*	*3.023*	*2.997*	*1.937*
Ho	1.817	2.991	2.883	2.001
	*1.565*	*3.070*	*2.954*	*1.972*
Y	1.798	3.211	2.988	1.964
	*1.568*	*2.909*	*3.174*	*1.958*
Er	1.892	2.964	2.778	2.122
	*1.544*	*3.020*	*2.901*	*2.011*

**Table 9:** X-ray diffraction pattern of BaLa_2_ZnO_5_ with *d* spacings (Å), Miller indices *h k l*, and integrated intensities *I* normalized to the value of 999 as the maximum

*d*	*I*	*h*	*k*	*l*	*d*	*I*	*h*	*k*	*l*	*d*	*I*	*h*	*k*	*l*
3.79885	53	0	0	2	3.73647	200	1	1	2	3.45491	20	2	0	0
2.98599	480	2	1	1	2.96806	999	2	0	2	2.89942	345	0	0	4
2.49345	22	1	1	4	2.44299	239	2	2	0	2.41379	540	2	1	3
2.25136	39	2	2	2	2.22096	20	2	0	4	2.18508	208	3	1	0
2.04473	31	3	1	2	1.93295	43	0	0	6	1.89080	18	3	2	1
1.86824	214	2	2	4	1.85508	207	2	1	5	1.79741	34	1	1	6
1.74502	114	3	1	4	1.72746	7	4	0	0	1.68688	178	2	0	6
1.65865	135	4	1	1	1.65556	104	4	0	2	1.62866	33	3	3	0
1.56799	97	3	3	2	1.54508	77	4	2	0	1.53762	131	4	1	3
1.49299	21	4	2	2	1.48403	19	4	0	4	1.46018	37	2	1	7
1.44971	44	0	0	8	1.44777	8	3	1	6	1.36356	82	4	2	4
1.35842	76	4	1	5	1.31957	27	5	1	2	1.28804	53	4	0	6
1.27534	24	5	2	1	1.24672	53	2	2	8	1.24549	66	3	3	6
1.22150	20	4	4	0	1.21780	41	5	2	3	1.20775	63	3	1	8M
1.20775	63	4	2	6M	1.19527	12	4	4	2	1.18936	36	2	1	9
1.18502	32	5	3	0	1.17823	42	4	1	7	1.16103	18	5	3	2
1.15164	56	6	0	0	1.13032	7	6	1	1+	1.12881	5	1	1	10+
1.12568	16	4	4	4	1.12278	27	5	2	5	1.10961	16	5	1	6
1.09948	42	2	0	10	1.09694	58	5	3	4	1.08286	17	3	3	8
1.07383	102	5	4	1M	1.07383	102	6	2	2M	1.07030	69	6	0	4
1.05721	45	4	2	8	1.03940	31	5	4	3	1.02155	32	4	1	9
1.01447	11	5	2	7	1.01028	8	5	3	6	0.99785	30	2	1	11
0.97843	24	5	4	5	0.96647	13	0	0	12	0.96361	14	5	5	2M
0.96361	14	7	1	2M	0.96289	18	4	0	10	0.95112	64	6	2	6
0.94564	70	7	2	1M	0.94564	70	6	4	2M	0.94472	30	3	3	10
0.93412	15	4	4	8	0.92176	30	7	2	3	0.91750	52	5	3	8
0.90951	31	6	4	4M	0.90951	31	5	2	9M	0.90424	20	5	4	7
0.90174	74	6	0	8	0.89870	25	2	2	12	0.89242	45	4	1	11
0.88388	28	3	1	12	0.88113	16	5	1	10	0.87844	32	7	2	5
0.87209	20	7	1	6M	0.87209	20	5	5	6M	0.86590	7	7	3	4
0.85852	46	6	4	6	0.85713	26	2	1	13	0.85452	100	8	1	1M
0.85452	100	7	4	1M	0.85452	100	8	0	2M	0.83794	31	8	2	0
0.83674	70	7	4	3M	0.83674	70	8	1	3M	0.82887	6	5	3	10
0.82735	16	5	4	9	0.82357	27	7	2	7					

**Table 10:** X-ray diffraction pattern of BaNd_2_ZnO_5_ with *d* spacings (Å), Miller indices *h k l*, and integrated intensities *I* normalized to the value of 999 as the maximumto the value of 999 as the maximum

*d*	*I*	*h*	*k*	*l*	*d*	*I*	*h*	*k*	*l*	*d*	*I*	*h*	*k*	*l*
5.77280	93	0	0	2	4.77989	8	1	1	0	3.68165	169	1	1	2
3.37989	20	2	0	0	2.92448	484	2	1	1	2.91674	999	2	0	2
2.88640	340	0	0	4	2.47085	31	1	1	4	2.38995	234	2	2	0
2.37733	541	2	1	3	2.20819	29	2	2	2	2.19493	17	2	0	4
2.13763	197	3	1	0	2.00461	18	3	1	2	1.92427	5	0	0	6
1.85059	9	3	2	1	1.84082	184	2	2	4	1.83504	206	2	1	5
1.78505	25	1	1	6	1.71784	104	3	1	4	1.68995	5	4	0	0
1.67224	171	2	0	6	1.62265	239	4	1	1M	1.62265	239	4	0	2M
1.59330	38	3	3	0	1.53587	121	3	3	2	1.51153	81	4	2	0
1.50833	141	4	1	3	1.49883	7	2	2	6	1.46224	29	4	2	2
1.45837	23	4	0	4	1.44789	47	2	1	7	1.44320	60	0	0	8
1.43016	6	3	1	6	1.39489	15	3	3	4	1.33904	80	4	2	4
1.33681	82	4	1	5	1.29207	25	5	1	2	1.26978	44	4	0	6
1.24791	22	5	2	1	1.23542	46	2	2	8	1.22722	61	3	3	6
1.19612	51	3	1	8	1.19497	16	4	4	0	1.19339	38	5	2	3
1.18866	13	4	2	6	1.18092	32	2	1	9	1.17017	11	4	4	2
1.16277	44	4	1	7	1.15929	33	5	3	0	1.13660	17	5	3	2
1.12663	51	6	0	0	1.12228	6	1	1	10	1.10602	10	6	1	1M
1.10602	10	6	0	2M	1.10409	15	4	4	4	1.10284	28	5	2	5
1.09232	62	2	0	10M	1.09232	62	5	1	6M	1.07577	60	5	3	4
1.06963	18	3	3	8	1.05104	104	5	4	1M	1.05104	104	6	2	2M
1.04952	69	6	0	4	1.04382	46	4	2	8	1.01809	30	5	4	3
1.01515	6	4	4	6	1.01032	34	4	1	9	0.99888	11	5	2	7
0.99301	11	5	3	6	0.99154	31	2	1	11	0.96213	15	0	0	12
0.96012	25	5	4	5	0.95332	18	4	0	10	0.94313	16	5	5	2M
0.94313	16	7	1	2M	0.93455	101	3	3	10M	0.93455	101	6	2	6M
0.92554	34	7	2	1	0.92529	44	6	4	2	0.92163	5	6	1	7
0.92041	17	4	4	8	0.91752	8	4	2	10	0.90381	48	5	3	8
0.90263	35	7	2	3	0.89720	13	5	2	9	0.89252	26	2	2	12
0.89157	10	6	4	4	0.88916	23	5	4	7	0.88802	84	6	0	8M
0.88802	84	7	3	0M	0.88397	40	4	1	11	0.87736	22	3	1	12
0.87066	14	5	1	10	0.86149	32	7	2	5	0.85615	17	7	1	6M
0.85615	17	5	5	6M	0.85211	27	2	1	13	0.84840	6	7	3	4
0.84273	47	6	4	6	0.83625	71	7	4	1M	0.83625	71	8	1	1M
0.83606	64	8	0	2+	0.82362	6	3	3	12					

**Table 11:** X-ray diffraction pattern of BaSm_2_ZnO_5_ with *d* spacings (Å), Miller indices *h k l*, and integrated intensities *I* normalized to the value of 999 as the maximum

*d*	*I*	*h*	*k*	*l*	*d*	*I*	*h*	*k*	*l*	*d*	*I*	*h*	*k*	*l*
6.29411	31	0	2	0	6.25287	49	1	1	0	4.74001	10	1	2	0
4.52364	66	1	0	1	3.67333	27	1	2	1	3.60226	19	2	0	0
3.46325	71	2	1	0	3.14705	129	0	4	0	3.12643	31	2	2	0
3.07636	999	1	3	1	2.97513	751	2	1	1	2.90609	592	0	0	2
2.88392	208	1	4	0	2.76742	105	0	4	1	2.75337	62	2	2	1
2.73323	21	2	3	0	2.58339	80	1	4	1	2.47363	157	2	3	1+
2.37636	98	1	5	0+	2.35896	24	3	1	0	2.26182	21	2	0	2
2.24373	114	3	2	0	2.22617	73	2	1	2	2.21951	54	3	0	1
2.19988	28	1	5	1	2.18579	57	3	1	1	2.13503	60	0	4	2
2.12855	17	2	2	2	2.09804	57	0	6	0	2.09318	16	3	2	1
2.04703	276	1	4	2	1.99099	115	2	3	2	1.97340	7	0	6	1
1.96195	23	3	3	1	1.94466	14	2	5	1	1.90914	142	3	4	0
1.90329	22	1	6	1	1.83978	51	1	5	2	1.81333	40	3	4	1M
1.81333	40	2	6	0M	1.80113	64	4	0	0	1.78297	14	4	1	0
1.77599	85	3	2	2	1.74478	49	1	7	0	1.73072	7	2	6	1
1.70877	145	1	3	3	1.70457	32	4	1	1	1.70106	27	0	6	2
1.69081	150	2	1	3	1.67111	46	1	7	1	1.66491	14	3	5	1
1.65554	11	1	6	2	1.64982	19	0	4	3	1.64683	12	2	2	3
1.60819	19	1	4	3	1.59562	137	3	4	2	1.58038	51	2	3	3M
1.58038	51	3	6	0M	1.57353	8	0	8	0	1.55065	104	2	7	1
1.53804	39	2	6	2M	1.53804	39	1	8	0M	1.53094	48	4	0	2
1.51919	47	4	1	2M	1.51919	47	0	8	1M	1.50923	75	4	4	1M
1.50923	75	3	0	3M	1.50168	7	1	5	3	1.49632	47	3	1	3M
1.49632	47	1	7	2M	1.48618	26	1	8	1	1.46554	16	3	2	3M
1.46554	16	4	5	0M	1.45305	94	0	0	4	1.43917	43	3	7	0M
1.43917	43	4	3	2M	1.42017	25	4	5	1M	1.42017	25	3	3	3M
1.40457	11	5	2	0	1.39857	10	5	0	1	1.39636	6	1	6	3
1.38811	24	3	6	2	1.38371	6	0	8	2	1.36661	13	4	6	0
1.35939	16	3	4	3M	1.35939	16	1	8	2M	1.33989	5	2	1	4
1.33627	8	1	9	1	1.33033	5	4	6	1	1.32681	90	5	3	1
1.31195	10	4	1	3	1.30808	6	4	5	2	1.29727	52	1	4	4M
1.29727	52	1	7	3M	1.28990	17	3	7	2	1.28366	43	3	8	1+
1.27223	6	2	9	1	1.26461	15	5	2	2	1.25882	8	0	10	0
1.25057	5	5	5	0	1.24315	28	4	7	1	1.23971	17	1	5	4
1.23778	57	2	7	3	1.23669	13	4	6	2	1.23030	14	0	10	1
1.22142	13	0	8	3	1.21963	28	3	2	4	1.21658	28	4	4	3
1.20424	16	1	8	3	1.20075	7	6	0	0	1.19893	5	3	8	2
1.19453	17	0	6	4	1.18835	5	2	10	0	1.18500	11	4	8	0
1.16846	13	4	5	3	1.16572	5	4	7	2	1.16371	7	5	6	1
1.16112	5	4	8	1	1.15624	48	3	4	4M	1.15624	48	5	0	3M
1.15475	21	0	10	2M	1.15475	21	6	3	0	1.14873	6	5	5	2
1.14054	16	1	10	2	1.13382	10	2	6	4	1.13091	30	4	0	4
1.12637	6	4	1	4	1.12447	7	5	7	0	1.12025	5	1	9	3
1.11656	22	1	7	4	1.11469	57	3	10	0M	1.11469	57	5	3	3M
1.10964	24	6	0	2	1.10964	24	1	11	1M	1.10694	41	1	3	5
1.10547	6	6	1	2	1.10400	7	5	7	1	1.10202	31	2	1	5
1.09994	8	2	10	2	1.09728	13	4	8	2	1.09062	36	2	11	0M
1.09062	36	0	4	5M	1.08870	17	3	8	3	1.08528	5	4	9	1
1.07815	7	1	4	5	1.07287	28	6	3	2	1.06962	21	2	3	5M
1.06962	21	3	6	4M	1.06751	6	0	8	4	1.06543	5	6	5	1
1.06365	15	4	7	3	1.05557	10	0	10	3	1.04870	16	5	7	2
1.04631	5	3	0	5	1.04232	16	3	1	5M	1.04232	16	6	6	0M
1.04095	19	3	10	2	1.03207	34	0	12	1+	1.02579	8	7	1	0M
1.02579	8	6	6	1M	1.02262	21	3	7	4	1.02112	56	2	11	2
1.01591	16	4	10	1	1.01345	18	7	0	1	1.01001	14	7	1	1M
1.01001	14	5	2	4M	0.99804	11	5	8	2	0.99550	12	4	6	4
0.98419	33	6	7	1	0.98098	28	6	6	2	0.97823	13	7	4	0
0.97624	7	1	11	3	0.97376	8	4	1	5	0.97240	15	5	7	3M
0.97240	15	4	10	2	0.96870	19	0	0	6	0.96737	12	1	7	5M
0.96737	12	7	1	2M	0.95284	10	4	11	1	0.95143	9	0	10	4
0.94786	6	5	5	4	0.94687	17	1	13	1	0.94523	5	7	3	2
0.94225	29	2	7	5	0.93564	12	5	10	1	0.93498	9	0	8	5
0.93282	27	2	1	6M	0.93282	27	4	4	5M	0.92714	31	1	8	5
0.92714	31	7	4	2M	0.92561	9	6	0	4	0.92325	5	2	13	1
0.92247	13	0	12	3	0.91989	6	2	10	4	0.91830	40	4	8	4M
0.91830	40	1	4	6M	0.91305	11	2	3	6	0.91070	13	4	10	3

**Table 12:** X-ray diffraction pattern of BaEu_2_ZnO_5_ with *d* spacings (Å), Miller indices *h k l*, and integrated intensities *I* normalized to the value of 999 as the maximum.

*d*	*I*	*h*	*k*	*l*	*d*	*I*	*h*	*k*	*l*	*d*	*I*	*h*	*k*	*l*
6.26795	33	0	2	0	6.22978	47	1	1	0	4.72157	15	1	2	0
4.50738	63	1	0	1	3.65943	11	1	2	1	3.58951	13	2	0	0
3.45083	54	2	1	0	3.13397	66	0	4	0	3.11489	19	2	2	0
3.06438	999	1	3	1	2.96443	815	2	1	1	2.89554	427	0	0	2
2.87222	194	1	4	0	2.75625	100	0	4	1	2.74324	67	2	2	1
2.72284	18	2	3	0	2.57312	92	1	4	1	2.46444	183	2	3	1+
2.36698	63	1	5	0	2.35056	15	3	1	0	2.25369	21	2	0	2
2.23562	125	3	2	0	2.21813	77	2	1	2	2.21163	56	3	0	1
2.19103	27	1	5	1	2.17799	62	3	1	1	2.12676	60	0	4	2
2.12077	13	2	2	2	2.08932	50	0	6	0	2.08560	14	3	2	1
2.03916	286	1	4	2	1.98358	92	2	3	2	1.95472	15	3	3	1
1.93704	5	2	5	1	1.90195	116	3	4	0	1.89557	18	1	6	1
1.83259	51	1	5	2	1.80624	36	3	4	1M	1.80624	36	2	6	0M
1.79476	66	4	0	0	1.77664	18	4	1	0	1.76956	113	3	2	2
1.73759	48	1	7	0	1.72385	7	2	6	1	1.70242	141	1	3	3
1.69851	32	4	1	1	1.69430	27	0	6	2	1.68469	138	2	1	3
1.66429	45	1	7	1	1.65855	12	3	5	1	1.64899	8	1	6	2
1.64360	17	0	4	3	1.64083	12	2	2	3	1.60214	21	1	4	3
1.58968	144	3	4	2	1.57444	54	2	3	3M	1.57444	54	3	6	0M
1.56699	8	0	8	0	1.54444	104	2	7	1	1.53196	48	2	6	2M
1.53196	48	1	8	0M	1.52548	52	4	0	2	1.51321	47	4	1	2M
1.51321	47	0	8	1M	1.50370	78	4	4	1M	1.50370	78	3	0	3M
1.49596	8	1	5	3	1.49178	17	3	1	3	1.48991	31	1	7	2
1.48010	30	1	8	1	1.45937	8	4	5	0	1.44777	82	0	0	4
1.43359	39	3	7	0M	1.43359	39	4	3	2M	1.41488	26	4	5	1M
1.41488	26	3	3	3M	1.40208	5	2	7	2	1.39955	12	5	2	0
1.39361	9	5	0	1	1.39097	5	1	6	3	1.38279	30	3	6	2
1.37812	7	0	8	2	1.36142	13	4	6	0	1.35425	10	3	4	3M
1.35425	10	1	8	2M	1.33504	7	2	1	4	1.33078	9	1	9	1
1.32203	88	5	3	1	1.31431	6	0	4	4	1.30725	14	4	1	3
1.30321	7	4	5	2	1.29282	30	1	4	4	1.29145	17	1	7	3
1.28490	20	3	7	2	1.27879	47	3	8	1+	1.26708	5	2	9	1
1.26008	13	5	2	2	1.25359	5	0	10	0	1.24596	6	5	5	0
1.23837	26	4	7	1	1.23644	5	1	9	2	1.23505	18	1	5	4+
1.23279	64	2	7	3+	1.22521	12	0	10	1	1.21660	15	0	8	3
1.21521	32	3	2	4	1.21212	30	4	4	3	1.20775	5	1	10	1
1.19950	15	1	8	3	1.19650	6	6	0	0	1.19424	5	3	8	2
1.18999	16	0	6	4	1.18349	7	2	10	0	1.18039	10	4	8	0
1.16413	14	4	5	3	1.15937	5	5	6	1	1.15661	5	4	8	1
1.15199	57	3	4	4+	1.15034	26	0	10	2M	1.15034	26	6	3	0M
1.14450	8	5	5	2	1.13591	15	1	10	2	1.12954	10	2	6	4
1.12685	32	4	0	4	1.12232	8	4	1	4	1.12022	6	5	7	0
1.11580	6	1	9	3	1.11228	23	1	7	4	1.11061	61	5	3	3+
1.10553	30	6	0	2M	1.10553	30	1	11	1M	1.10289	38	1	3	5
1.10154	8	6	1	2	1.09983	7	5	7	1	1.09802	34	2	1	5
1.09552	9	2	10	2	1.09306	14	4	8	2	1.08899	5	6	2	2
1.08618	37	2	11	0+	1.08449	20	3	8	3	1.08103	5	4	9	1
1.06901	26	6	3	2	1.06565	20	2	3	5M	1.06565	20	3	6	4M
1.06338	5	0	8	4	1.06154	5	6	5	1	1.05963	18	4	7	3
1.05135	8	0	10	3	1.04476	16	5	7	2	1.04265	11	6	4	2M
1.04265	11	3	0	5M	1.03851	18	3	1	5M	1.03851	18	6	6	0M
1.03682	17	3	10	2	1.02793	31	0	12	1+	1.02208	10	7	1	0M
1.02208	10	6	6	1M	1.01875	24	3	7	4	1.01700	62	2	11	2
1.01304	5	3	11	1	1.01191	14	4	10	1	1.00986	18	7	0	1
1.00641	18	7	1	1M	1.00641	18	5	2	4M	0.99425	10	5	8	2
0.99179	12	4	6	4	0.98052	31	6	7	1	0.97736	32	6	6	2
0.97471	14	7	4	0	0.97212	11	1	11	3M	0.97212	11	3	8	4M
0.97025	7	4	1	5	0.96864	18	5	7	3M	0.96864	18	4	10	2M
0.96518	25	0	0	6	0.96378	15	7	1	2M	0.96378	15	1	7	5M
0.94906	11	4	11	1	0.94769	8	0	10	4	0.94500	5	5	9	2
0.94438	6	5	5	4	0.94296	14	1	13	1	0.94185	6	7	3	2
0.93870	27	2	7	5	0.93176	20	5	10	1M	0.93176	20	0	8	5M
0.92942	22	2	1	6M	0.92942	22	4	4	5M	0.92374	31	7	4	2M
0.92374	31	1	8	5M	0.92235	14	0	4	6M	0.92235	14	6	0	4M
0.91875	14	0	12	3	0.91630	7	2	10	4	0.91490	38	1	4	6M
0.91490	38	4	8	4M	0.90972	9	2	3	6	0.90798	5	3	11	3

**Table 13:** X-ray diffraction pattern of BaGd_2_ZnO_5_ with *d* spacings (Å), Miller indices *h k l*, and integrated intensities *I* normalized to the value of 999 as the maximum

*d*	*I*	*h*	*k*	*l*	*d*	*I*	*h*	*k*	*l*	*d*	*I*	*h*	*k*	*l*
6.24700	30	0	2	0	6.21045	51	1	1	0	4.49408	62	1	0	1
3.64814	10	1	2	1	3.57865	15	2	0	0	3.44031	62	2	1	0
3.12350	85	0	4	0	3.10523	31	2	2	0	3.05469	999	1	3	1
2.95550	847	2	1	1	2.88713	525	0	0	2	2.86276	217	1	4	0
2.74731	112	0	4	1	2.73485	74	2	2	1	2.71424	22	2	3	0
2.56485	93	1	4	1	2.45665	156	2	3	1+	2.35915	77	1	5	0
2.35322	5	2	4	0	2.34343	19	3	1	0	2.24704	27	2	0	2
2.22876	132	3	2	0	2.21156	84	2	1	2	2.20497	56	3	0	1
2.18391	30	1	5	1	2.17920	6	2	4	1	2.17142	69	3	1	1
2.12015	66	0	4	2	2.11441	18	2	2	2	2.08173	77	0	6	0M
2.08173	77	3	2	1M	2.03284	318	1	4	2	1.99943	5	1	6	0
1.97755	115	2	3	2	1.95885	5	0	6	1	1.94870	17	3	3	1
1.93084	7	2	5	1	1.89597	126	3	4	0	1.88937	20	1	6	1
1.85872	7	1	0	3	1.82683	54	1	5	2	1.80051	42	3	4	1M
1.80051	42	2	6	0M	1.78933	72	4	0	0	1.77125	18	4	1	0
1.76424	113	3	2	2	1.73182	58	1	7	0	1.71828	12	2	6	1
1.69734	157	1	3	3	1.69338	36	4	1	1	1.68889	26	0	6	2
1.67974	147	2	1	3	1.65882	47	1	7	1	1.65332	12	3	5	1
1.64374	9	1	6	2	1.63862	21	0	4	3	1.63597	14	2	2	3
1.59730	22	1	4	3+	1.58480	153	3	4	2	1.56961	54	2	3	3M
1.56961	54	3	6	0M	1.56175	10	0	8	0	1.53941	102	2	7	1
1.52708	46	2	6	2M	1.52708	46	1	8	0M	1.52092	53	4	0	2
1.50977	18	4	1	2	1.50758	35	0	8	1	1.49911	79	4	4	1M
1.49911	79	3	0	3M	1.49137	8	1	5	3	1.48737	21	3	1	3
1.48513	34	1	7	2	1.47521	35	1	8	1	1.45480	11	4	5	0
1.44357	101	0	0	4	1.42903	46	3	7	0M	1.42903	46	4	3	2M
1.41054	31	4	5	1M	1.41054	31	3	3	3M	1.39761	7	2	7	2
1.39530	13	5	2	0	1.38940	11	5	0	1	1.38666	5	1	6	3
1.37845	34	3	6	2	1.37365	7	0	8	2	1.35712	15	4	6	0+
1.35072	8	3	4	3	1.33113	8	2	1	4	1.32638	9	1	9	1
1.31799	95	5	3	1	1.31039	6	0	4	4	1.30336	12	4	1	3
1.29919	9	4	5	2	1.29425	6	2	9	0	1.28896	35	1	4	4
1.28740	20	1	7	3	1.28083	18	3	7	2	1.27578	10	5	1	2
1.27446	36	2	3	4M	1.27446	36	3	8	1M	1.26322	10	4	7	0M
1.26322	10	2	9	1M	1.25628	16	5	2	2	1.24940	8	0	10	0
1.24209	9	5	5	0	1.23445	30	4	7	1	1.23134	19	1	5	4+
1.22913	50	2	7	3+	1.22820	13	4	6	2	1.22114	14	0	10	1
1.21275	16	0	8	3	1.21162	34	3	2	4	1.20845	32	4	4	3
1.20375	5	1	10	1	1.19570	17	1	8	3	1.19288	6	6	0	0
1.19043	5	3	8	2	1.18637	17	0	6	4	1.17958	7	2	10	0
1.17661	9	4	8	0	1.16058	18	4	5	3	1.15763	5	4	7	2
1.14863	7	5	0	3	1.14855	50	3	4	4+	1.14670	25	6	3	0M
1.14670	25	0	10	2M	1.14098	8	5	5	2	1.13220	17	1	10	2
1.12610	12	2	6	4	1.12352	31	4	0	4	1.11900	8	4	1	4
1.11669	8	5	7	0	1.11225	6	1	9	3	1.10886	25	1	7	4+
1.10729	58	5	3	3	1.10681	8	3	10	0	1.10249	20	6	0	2
1.10119	7	1	11	1	1.09965	45	1	3	5	1.09822	8	6	1	2
1.09638	7	5	7	1	1.09482	37	2	1	5+	1.09196	9	2	10	2
1.08960	13	4	8	2	1.08260	38	2	11	0+	1.08109	20	3	8	3
1.07756	6	4	9	1	1.07099	8	1	4	5	1.06578	27	6	3	2
1.06247	24	2	3	5M	1.06247	24	3	6	4M	1.05828	6	6	5	1
1.05635	17	4	7	3	1.04797	11	0	10	3	1.04150	14	5	7	2
1.03508	13	6	6	0	1.03347	19	3	10	2	1.02464	34	0	12	1+
1.01893	10	7	1	0M	1.01893	10	6	6	1M	1.01563	21	3	7	4
1.01368	67	2	11	2	1.00973	5	3	11	1	1.00864	15	4	10	1+
1.00681	20	7	0	1	1.00340	22	7	1	1M	1.00340	22	5	2	4M
0.99298	5	7	3	0	0.99113	12	5	8	2	0.98878	11	4	6	4
0.97746	30	6	7	1	0.97435	31	6	6	2	0.97173	13	7	4	0
0.96899	11	1	11	3M	0.96899	11	3	8	4M	0.96740	10	4	1	5
0.96556	20	5	7	3M	0.96556	20	4	10	2M	0.96238	22	0	0	6
0.96087	15	7	1	2M	0.96087	15	1	7	5M	0.95276	12	4	9	3M
0.95276	12	7	2	2M	0.95083	6	4	7	4	0.94598	12	4	11	1
0.94471	9	0	10	4	0.94178	16	5	9	2M	0.94178	16	5	5	4M
0.93976	20	1	13	1M	0.93976	20	6	5	3M	0.93900	7	7	3	2
0.93585	32	2	7	5	0.92884	25	5	10	1M	0.92884	25	0	8	5M
0.92668	26	2	1	6M	0.92668	26	4	4	5M	0.92096	27	7	4	2+

**Table 14:** X-ray diffraction pattern of BaDy_2_ZnO_5_ with *d* spacings (Å), Miller indices *h k l*, and integrated intensities *I* normalized to the value of 999 as the maximum

*d*	*I*	*h*	*k*	*l*	*d*	*I*	*h*	*k*	*l*	*d*	*I*	*h*	*k*	*l*
6.19234	25	0	2	0	6.15523	32	1	1	0	4.66487	7	1	2	0
4.45727	66	1	0	1	3.61756	13	1	2	1	3.54666	12	2	0	0
3.40961	58	2	1	0	3.09617	63	0	4	0	3.07761	24	2	2	0
3.02875	999	1	3	1	2.93007	827	2	1	1	2.86490	515	0	0	2
2.83763	233	1	4	0	2.72392	112	0	4	1	2.71126	71	2	2	1
2.69019	18	2	3	0	2.54287	105	1	4	1	2.44127	12	1	2	2
2.43515	160	2	3	1	2.33846	89	1	5	0	2.33244	6	2	4	0
2.32250	18	3	1	0	2.22863	29	2	0	2	2.20889	138	3	2	0
2.19340	96	2	1	2	2.18566	61	3	0	1	2.16031	7	2	4	1
2.15240	73	3	1	1	2.10280	60	0	4	2	2.09696	17	2	2	2
2.06345	75	0	6	0M	2.06345	75	3	2	1M	2.01608	315	1	4	2
1.98191	7	1	6	0	1.96111	118	2	3	2	1.94195	5	0	6	1
1.93164	18	3	3	1	1.91407	9	2	5	1	1.87916	134	3	4	0
1.87302	21	1	6	1	1.81159	53	1	5	2	1.78468	39	3	4	1M
1.78468	39	2	6	0M	1.77333	60	4	0	0	1.75543	20	4	1	0
1.74931	114	3	2	2	1.71665	61	1	7	0	1.70333	12	2	6	1
1.68386	192	1	3	3	1.67842	42	4	1	1	1.67471	26	0	6	2
1.66631	171	2	1	3	1.64443	48	1	7	1	1.63885	11	3	5	1
1.62990	10	1	6	2	1.62553	19	0	4	3	1.62283	17	2	2	3
1.58446	22	1	4	3+	1.57130	138	3	4	2	1.55735	41	2	3	3
1.55496	23	3	6	0	1.54808	7	0	8	0	1.52600	99	2	7	1
1.51406	49	2	6	2M	1.51406	49	1	8	0M	1.50784	51	4	0	2
1.49679	17	4	1	2	1.49450	30	0	8	1	1.48607	73	4	4	1M
1.48607	73	3	0	3M	1.47925	8	1	5	3	1.47518	21	3	1	3
1.47253	35	1	7	2	1.46239	32	1	8	1	1.44189	10	4	5	0
1.43245	75	0	0	4	1.41650	44	3	7	0M	1.41650	44	4	3	2M
1.39830	26	4	5	1+	1.38568	7	2	7	2	1.38284	12	5	2	0
1.37708	10	5	0	1	1.37527	6	1	6	3+	1.36663	28	3	6	2
1.36196	5	0	8	2	1.34510	11	4	6	0	1.33951	5	3	4	3
1.33753	5	1	8	2	1.32063	6	2	1	4	1.31484	12	1	9	1
1.30632	86	5	3	1	1.30372	5	2	6	3	1.30005	7	0	4	4
1.29517	5	3	8	0	1.29245	13	4	1	3	1.28796	8	4	5	2
1.27875	34	1	4	4	1.27673	20	1	7	3	1.26982	19	3	7	2
1.26459	13	5	1	2M	1.26459	13	2	3	4M	1.26330	34	3	8	1
1.25216	9	4	7	0M	1.25216	9	2	9	1M	1.24535	16	5	2	2
1.23847	8	0	10	0	1.23105	6	5	5	0	1.22359	25	4	7	1
1.22157	23	1	9	2M	1.22157	23	1	5	4M	1.21888	42	2	7	3
1.21757	11	4	6	2	1.21051	11	0	10	1	1.20211	47	0	8	3M
1.20211	47	3	2	4M	1.19827	22	4	4	3	1.18572	15	1	8	3
1.18222	7	6	0	0	1.18017	6	3	8	2	1.17683	17	0	6	4+
1.16923	6	2	10	0	1.16622	5	4	8	0	1.15078	15	4	5	3
1.14761	6	4	7	2	1.14554	5	5	6	1	1.14279	5	4	8	1
1.13921	44	3	4	4	1.13858	13	5	0	3M	1.13858	13	6	2	1M
1.13669	23	0	10	2M	1.13669	23	6	3	0M	1.13105	8	5	5	2
1.12247	16	1	10	2	1.11694	10	2	6	4	1.11432	27	4	0	4
1.10983	9	4	1	4	1.10445	5	6	4	0	1.10290	6	1	9	3
1.09984	23	1	7	4+	1.09777	59	5	3	3M	1.09777	59	3	10	0M
1.09283	21	6	0	2	1.09106	45	1	3	5	1.08860	8	6	1	2
1.08634	43	5	7	1M	1.08634	43	2	1	5M	1.08255	11	2	10	2
1.08015	10	4	8	2	1.07609	7	6	2	2+	1.07311	30	2	11	0
1.07195	20	3	8	3	1.06810	7	4	9	1	1.06258	7	1	4	5+
1.05644	16	6	3	2	1.05387	21	2	3	5M	1.05387	21	3	6	4M
1.04890	6	6	5	1	1.04737	17	4	7	3	1.04591	5	5	8	0
1.03950	17	1	8	4M	1.03950	17	0	10	3M	1.03243	10	5	7	2
1.03052	6	6	4	2	1.02767	6	3	1	5	1.02587	13	6	6	0
1.02453	14	3	10	2	1.01576	28	0	12	1+	1.00988	13	7	1	0M
1.00988	13	6	6	1M	1.00723	21	3	7	4	1.00492	69	2	11	2
0.99979	14	4	10	1	0.99785	13	7	0	1+	0.99479	19	5	2	4M
0.99479	19	7	1	1M	0.98249	10	5	8	2	0.98055	11	4	6	4
0.96881	26	6	7	1	0.96582	30	6	6	2	0.96306	10	7	4	0
0.96070	6	3	8	4	0.95959	7	4	1	5	0.95723	18	5	7	3M
0.95723	18	4	10	2M	0.95511	24	2	9	4M	0.95511	24	0	0	6M
0.95291	16	1	7	5M	0.95291	16	7	1	2M	0.94481	5	4	9	3
0.94416	5	7	2	2	0.94289	5	4	7	4	0.93768	12	4	11	1
0.93686	7	0	10	4	0.93373	13	5	9	2M	0.93373	13	5	5	4M
0.93163	14	1	13	1+	0.93074	5	7	3	2	0.92830	22	2	7	5

**Table 15:** X-ray diffraction pattern of BaHo_2_ZnO_5_ with *d* spacings (Å), Miller indices *h k l*, and integrated intensities *I* normalized to the value of 999 as the maximum

*d*	*I*	*h*	*k*	*l*	*d*	*I*	*h*	*k*	*l*	*d*	*I*	*h*	*k*	*l*
6.17101	15	0	2	0	6.13550	17	1	1	0	4.44320	29	1	0	1
3.60579	11	1	2	1	3.53558	16	2	0	0	3.39886	65	2	1	0
3.08551	83	0	4	0	3.06775	22	2	2	0	3.01872	999	1	3	1
2.92082	820	2	1	1	2.85580	489	0	0	2	2.82800	222	1	4	0
2.71471	90	0	4	1	2.70259	79	2	2	1	2.68142	8	2	3	0
2.58908	9	1	1	2	2.53436	127	1	4	1	2.43343	9	1	2	2
2.42724	164	2	3	1	2.33049	99	1	5	0	2.32472	6	2	4	0
2.31521	18	3	1	0	2.22160	32	2	0	2	2.20190	156	3	2	0
2.18646	88	2	1	2	2.17881	53	3	0	1	2.15778	30	1	5	1
2.15320	7	2	4	1	2.14563	86	3	1	1	2.09586	87	0	4	2
2.09027	22	2	2	2	2.05654	87	0	6	0M	2.05654	87	3	2	1M
2.00945	301	1	4	2	1.97513	7	1	6	0	1.95479	96	2	3	2
1.93532	5	0	6	1	1.92545	13	3	3	1	1.90771	13	2	5	1
1.87306	113	3	4	0	1.86667	20	1	6	1	1.80558	69	1	5	2
1.77879	47	3	4	1M	1.77879	47	2	6	0M	1.76779	66	4	0	0
1.74993	26	4	1	0	1.74376	141	3	2	2	1.71077	57	1	7	0
1.69763	14	2	6	1	1.67844	163	1	3	3	1.67316	36	4	1	1
1.66910	24	0	6	2	1.66103	143	2	1	3	1.63883	54	1	7	1
1.63349	13	3	5	1	1.62446	11	1	6	2	1.62025	15	0	4	3
1.61766	14	2	2	3	1.57932	26	1	4	3	1.56623	132	3	4	2
1.55236	37	2	3	3	1.54982	21	3	6	0	1.54275	7	0	8	0
1.52087	87	2	7	1	1.50904	51	2	6	2M	1.50904	51	1	8	0M
1.50311	44	4	0	2	1.49208	22	4	1	2	1.48938	36	0	8	1
1.48132	60	4	4	1M	1.48132	60	3	0	3M	1.47441	7	1	5	3
1.47052	29	3	1	3	1.46759	32	1	7	2	1.45740	29	1	8	1
1.43723	12	4	5	0	1.42790	88	0	0	4	1.41184	49	3	7	0M
1.41184	49	4	3	2	1.39378	30	4	5	1+	1.38674	5	2	5	3
1.38106	11	2	7	2	1.37849	15	5	2	0	1.37277	11	5	0	1
1.37074	6	1	6	3+	1.36216	36	3	6	2	1.34071	14	4	6	0
1.33521	8	3	4	3	1.33302	6	1	8	2	1.31645	6	2	1	4
1.31035	10	1	9	1	1.30219	85	5	3	1	1.29945	5	2	6	3
1.29586	7	0	4	4	1.29083	5	3	8	0	1.28838	14	4	1	3
1.28381	9	4	5	2	1.27464	30	1	4	4	1.27250	19	1	7	3
1.26563	15	3	7	2	1.26060	10	5	1	2M	1.26060	10	2	3	4M
1.25908	24	3	8	1	1.24796	12	4	7	0M	1.24796	12	2	9	1M
1.24143	14	5	2	2	1.23420	8	0	10	0	1.22710	6	5	5	0
1.21958	24	4	7	1	1.21759	24	1	9	2M	1.21759	24	1	5	4M
1.21460	47	2	7	3M	1.21460	47	4	6	2M	1.20636	11	0	10	1
1.19822	49	0	8	3M	1.19822	49	3	2	4M	1.19445	23	4	4	3
1.18177	17	1	8	3	1.17853	6	6	0	0	1.17626	5	3	8	2
1.17299	15	0	6	4	1.16525	7	2	10	0	1.16236	7	4	8	0
1.15739	6	6	2	0M	1.15739	6	1	6	4M	1.14708	16	4	5	3
1.14386	6	4	7	2	1.13552	46	3	4	4M	1.13552	46	5	0	3M
1.13294	20	6	3	0M	1.13294	20	0	10	2M	1.12743	7	5	5	2
1.11866	13	1	10	2	1.11332	10	2	6	4	1.11080	26	4	0	4
1.10633	7	4	1	4	1.09921	5	1	9	3	1.09623	19	1	7	4+
1.09336	6	3	10	0+	1.08941	17	6	0	2	1.08758	35	1	3	5
1.08519	8	6	1	2	1.08286	36	5	7	1M	1.08286	36	2	1	5M
1.07889	9	2	10	2	1.07669	9	4	8	2	1.07282	5	6	2	2
1.06944	26	2	11	0	1.06841	16	3	8	3	1.06455	6	4	9	1
1.06141	5	2	9	3	1.05908	9	1	4	5+	1.05311	19	6	3	2
1.05099	13	2	3	5+	1.05014	12	3	6	4	1.04555	5	6	5	1
1.04396	14	4	7	3	1.03661	5	1	8	4	1.03563	8	0	10	3
1.02908	7	5	7	2	1.02441	5	3	1	5	1.02258	10	6	6	0
1.02110	12	3	10	2	1.01234	30	0	12	1+	1.00658	6	6	6	1
1.00396	20	3	7	4	1.00152	56	2	11	2+	0.99645	12	4	10	1+
0.99452	17	7	0	1+	0.99171	22	5	2	4+	0.98102	5	7	3	0
0.97928	11	5	8	2	0.97740	11	4	6	4	0.96569	22	6	7	1
0.96273	30	6	6	2	0.96002	10	7	4	0	0.95756	5	3	8	4
0.95655	8	4	1	5	0.95453	5	5	7	3	0.95385	11	4	10	2
0.95193	18	0	0	6	0.94984	18	1	7	5M	0.94984	18	7	1	2M
0.94731	5	4	11	0	0.94148	11	4	9	3M	0.94148	11	7	2	2M
0.93983	6	4	7	4	0.93454	13	4	11	1	0.93374	9	0	10	4
0.93070	17	5	9	2M	0.93070	17	5	5	4M	0.92827	25	1	13	1+
0.92528	20	2	7	5	0.91791	21	0	8	5M	0.91791	21	5	10	1M
0.91631	18	2	1	6M	0.91631	18	4	4	5M	0.91003	23	7	4	2+

**Table 16:** X-ray diffraction pattern of BaY_2_ZnO_5_ with *d* spacings (Å), Miller indices *h k l*, and integrated intensities *I* normalized to the value of 999 as the maximum

*d*	*I*	*h*	*k*	*l*	*d*	*I*	*h*	*k*	*l*	*d*	*I*	*h*	*k*	*l*
6.16838	7	0	2	0	6.13423	32	1	1	0	4.18582	15	0	2	1M
4.18582	15	1	1	1M	3.60450	6	1	2	1	3.55471	11	1	3	0
3.53510	14	2	0	0	3.39833	57	2	1	0	3.08419	112	0	4	0
3.06712	40	2	2	0	3.01758	999	1	3	1	2.92015	831	2	1	1
2.85454	429	0	0	2	2.82693	202	1	4	0	2.71354	33	0	4	1
2.70189	144	2	2	1	2.58804	46	1	1	2	2.53336	161	1	4	1
2.43245	10	1	2	2	2.42654	163	2	3	1	2.32957	115	1	5	0+
2.31487	17	3	1	0	2.22180	53	1	3	2M	2.22180	53	2	0	2M
2.20152	194	3	2	0	2.18575	101	2	1	2	2.17842	25	3	0	1
2.15692	33	1	5	1	2.14523	90	3	1	1	2.09495	121	0	4	2
2.08958	24	2	2	2	2.05563	82	0	6	0M	2.05563	82	3	2	1
2.00863	328	1	4	2	1.95412	74	2	3	2	1.92500	9	3	3	1
1.90705	26	2	5	1	1.87261	135	3	4	0	1.86591	19	1	6	1
1.80483	96	1	5	2	1.77828	70	3	4	1M	1.77828	70	2	6	0M
1.76755	84	4	0	0	1.74968	37	4	1	0	1.74329	171	3	2	2
1.71007	49	1	7	0	1.69702	21	2	6	1	1.67773	166	1	3	3
1.67288	37	4	1	1	1.66838	23	0	6	2	1.66041	159	2	1	3
1.63816	64	1	7	1	1.63302	17	3	5	1	1.62383	15	4	3	0M
1.62383	15	1	6	2M	1.61954	6	0	4	3	1.61705	29	2	2	3
1.57842	48	1	4	3M	1.57842	48	2	7	0M	1.56576	141	3	4	2
1.55177	43	2	3	3	1.54935	25	3	6	0	1.52030	75	2	7	1
1.50829	59	2	6	2M	1.50829	59	1	8	0M	1.50278	37	4	0	2
1.49175	34	4	1	2	1.48874	48	0	8	1	1.48100	45	4	4	1M
1.48100	45	3	0	3M	1.47379	8	1	5	3	1.47004	22	3	1	3
1.46697	19	1	7	2	1.45680	30	1	8	1	1.43970	8	3	2	3
1.43689	14	4	5	0	1.42727	78	0	0	4	1.41144	61	4	3	2M
1.41144	61	3	7	0M	1.39344	32	4	5	1	1.38053	24	2	7	2
1.37829	19	5	2	0	1.37257	12	5	0	1	1.37016	6	1	6	3+
1.36415	8	5	1	1	1.36170	40	3	6	2	1.34036	13	4	6	0
1.33476	11	3	4	3	1.33246	9	1	8	2	1.31592	6	2	1	4
1.30980	14	1	9	1	1.30196	84	5	3	1	1.29894	7	2	6	3
1.29530	12	0	4	4	1.29040	8	3	8	0	1.28802	16	4	1	3
1.28346	12	4	5	2	1.27804	7	2	9	0	1.27409	28	1	4	4
1.27197	25	1	7	3	1.26956	5	3	5	3	1.26519	17	3	7	2
1.26047	6	5	1	2	1.25865	27	3	8	1	1.24760	16	4	7	0M
1.24760	16	2	9	1M	1.24118	18	5	2	2	1.23368	5	0	10	0
1.22685	7	5	5	0	1.21923	26	4	7	1	1.21707	33	1	9	2M
1.21707	33	1	5	4M	1.21412	44	2	7	3M	1.21412	44	4	6	2M
1.20584	11	0	10	1	1.19778	63	0	8	3M	1.19778	63	3	2	4M
1.19409	18	4	4	3	1.18127	15	1	8	3	1.17837	7	6	0	0
1.17584	8	3	8	2	1.17248	17	0	6	4	1.16646	5	2	9	2
1.16479	8	2	10	0	1.16201	6	4	8	0	1.14673	17	4	5	3
1.14351	8	4	7	2	1.14158	7	5	6	1	1.13867	5	4	8	1
1.13512	49	3	4	4M	1.13512	49	5	0	3M	1.13436	10	6	2	1
1.13255	21	6	3	0M	1.13255	21	0	10	2M	1.12715	10	5	5	2
1.11819	13	1	10	2	1.11286	12	2	6	4	1.11044	29	4	0	4
1.10597	12	4	1	4	1.09874	8	1	9	3	1.09576	20	1	7	4
1.09410	52	5	3	3	1.08921	18	6	0	2	1.08711	36	1	3	5
1.08499	7	6	1	2	1.08244	48	5	7	1M	1.08244	48	2	1	5M
1.07626	7	4	8	2	1.07306	14	3	10	1+	1.07306	14	6	2	2+
1.07007	6	2	2	5	1.06902	28	2	11	0	1.06802	16	3	8	3
1.06421	9	4	9	1	1.05857	14	1	4	5M	1.05857	14	2	7	4M
1.05290	19	6	3	2	1.05007	25	2	3	5M	1.05007	25	3	6	4M
1.04362	20	4	7	3	1.03617	8	1	8	4	1.03519	8	0	10	3
1.02704	5	6	4	2	1.02402	6	3	1	5	1.02237	13	6	6	0
1.02072	14	3	10	2	1.01198	31	4	5	4+	1.01198	31	0	12	1+
1.00650	11	7	1	0M	1.00650	11	6	6	1M	1.00357	23	3	7	4
1.00112	61	2	11	2	0.99612	11	4	10	1	0.99458	13	7	0	1
0.99143	21	5	2	4M	0.99143	21	7	1	1M	0.98889	6	6	2	3
0.97900	13	5	8	2	0.97706	12	4	6	4	0.97488	5	3	4	5
0.96547	25	6	7	1	0.96250	37	6	6	2	0.95987	9	7	4	0
0.95719	7	3	8	4	0.95622	7	4	1	5	0.95378	20	5	7	3M
0.95378	20	4	10	2M	0.95165	25	2	9	4M	0.95165	25	0	0	6M
0.94950	18	1	7	5M	0.94950	18	7	1	2M	0.94121	12	4	9	3M
0.94121	12	7	2	2M	0.93950	7	4	7	4	0.93422	9	4	11	1
0.93334	7	0	10	4	0.93042	18	5	5	4M	0.93042	18	3	12	1M

**Table 17:** X-ray diffraction pattern of BaEr_2_ZnO_5_ with *d* spacings (Å), Miller indices *h k l*, and integrated intensities *I* normalized to the value of 999 as the maximum

*d*	*I*	*h*	*k*	*l*	*d*	*I*	*h*	*k*	*l*	*d*	*I*	*h*	*k*	*l*
6.14905	17	0	2	0	6.11312	42	1	1	0	4.63267	8	1	2	0
4.42851	70	1	0	1	4.17113	5	0	2	1M	4.17113	5	1	1	1M
3.59356	13	1	2	1	3.52257	17	2	0	0	3.38640	73	2	1	0
3.07453	68	0	4	0	3.05656	21	2	2	0	3.00833	999	1	3	1
2.91057	809	2	1	1	2.84705	596	0	0	2	2.81788	216	1	4	0
2.70535	134	0	4	1	2.69308	63	2	2	1	2.67169	24	2	3	0
2.52554	106	1	4	1	2.42561	9	1	2	2	2.41869	152	2	3	1
2.32217	81	1	5	0	2.31633	5	2	4	0	2.30670	18	3	1	0
2.21425	31	2	0	2	2.19384	150	3	2	0	2.17921	94	2	1	2
2.17099	68	3	0	1	2.15023	33	1	5	1	2.14560	8	2	4	1
2.13794	81	3	1	1	2.08896	69	0	4	2	2.08330	20	2	2	2
2.04922	72	0	6	0M	2.04922	72	3	2	1M	2.00278	305	1	4	2
1.96808	9	1	6	0	1.94822	140	2	3	2	1.92854	7	0	6	1
1.91855	23	3	3	1	1.90096	12	2	5	1	1.86625	141	3	4	0
1.86011	24	1	6	1	1.83269	5	1	0	3	1.79950	55	1	5	2
1.77241	43	3	4	1M	1.77241	43	2	6	0M	1.76129	70	4	0	0
1.74350	27	4	1	0	1.73777	120	3	2	2	1.70467	63	1	7	0
1.69162	14	2	6	1	1.67310	168	1	3	3	1.66710	36	4	1	1
1.66344	25	0	6	2	1.65570	144	2	1	3	1.63306	51	1	7	1
1.62765	11	3	5	1	1.61893	12	1	6	2	1.61506	23	0	4	3
1.61244	12	2	2	3	1.57423	22	1	4	3	1.56081	153	3	4	2
1.54731	40	2	3	3	1.54422	21	3	6	0	1.53726	8	0	8	0
1.51548	108	2	7	1	1.50416	38	2	6	2	1.50192	7	1	8	0
1.49784	55	4	0	2	1.48685	16	4	1	2	1.48413	37	0	8	1
1.47607	82	3	0	3M	1.47607	82	4	4	1M	1.46944	10	1	5	3M
1.46944	10	2	4	3M	1.46565	20	3	1	3	1.46255	39	1	7	2
1.45225	30	1	8	1	1.43539	5	3	2	3	1.43200	13	4	5	0
1.42352	93	0	0	4	1.40679	45	4	3	2M	1.40679	45	3	7	0M
1.39987	5	5	1	0	1.38878	31	3	3	3M	1.38878	31	4	5	1M
1.37628	6	2	7	2	1.37343	13	5	2	0	1.36777	11	5	0	1
1.36615	8	1	6	3M	1.36615	8	3	7	1M	1.35741	32	3	6	2
1.35267	7	0	8	2	1.33585	15	4	6	0+	1.33132	9	5	3	0M
1.33132	9	3	4	3M	1.32841	5	1	8	2	1.31229	7	2	1	4
1.30571	7	1	9	1	1.29746	92	5	3	1	1.29178	6	0	4	4
1.28400	13	4	1	3	1.27929	11	4	5	2	1.27060	37	1	4	4
1.26825	19	1	7	3	1.26121	18	3	7	2	1.25625	16	2	3	4M
1.25625	16	5	1	2M	1.25459	32	3	8	1	1.24323	6	2	9	1
1.23702	15	5	2	2	1.22981	6	0	10	0	1.22262	6	5	5	0
1.21520	24	4	7	1	1.21364	15	1	5	4	1.21076	44	2	7	3
1.20934	11	4	6	2	1.20209	17	0	10	1	1.19430	45	0	8	3M
1.19430	45	3	2	4M	1.19036	29	4	4	3	1.17778	15	1	8	3
1.16915	19	0	6	4M	1.16915	19	6	1	0M	1.16108	5	2	10	0
1.15817	6	4	8	0	1.14315	20	4	5	3	1.13982	6	4	7	2
1.13771	6	5	6	1M	1.13771	6	2	10	1M	1.13493	5	4	8	1
1.13184	46	3	4	4+	1.12890	21	0	10	2M	1.12890	21	6	3	0M
1.12342	6	5	5	2+	1.11476	15	1	10	2	1.10968	9	2	6	4
1.10713	29	4	0	4	1.10267	7	4	1	4	1.09919	7	5	7	0
1.09547	5	1	9	3	1.09265	25	1	7	4+	1.09059	54	5	3	3
1.08949	8	4	2	4M	1.08949	8	3	10	0M	1.08550	17	6	0	2
1.08416	48	1	3	5M	1.08416	48	1	11	1M	1.08129	9	6	1	2
1.07939	44	2	1	5M	1.07939	44	5	7	1M	1.07512	8	2	10	2
1.07280	11	4	8	2	1.06897	5	6	2	2	1.06766	9	0	4	5M
1.06766	9	2	2	5M	1.06563	32	2	11	0	1.06475	20	3	8	3
1.05585	6	1	4	5	1.04933	21	6	3	2	1.04762	9	2	3	5
1.04683	14	2	11	1M	1.04683	14	3	6	4M	1.04036	15	4	7	3
1.03210	10	0	10	3	1.02542	14	5	7	2	1.02115	7	3	1	5
1.01885	12	6	6	0	1.01751	15	3	10	2	1.00957	7	4	5	4
1.00856	22	0	12	1M	1.00856	22	4	10	0M	1.00299	8	7	1	0M
1.00299	8	6	6	1M	1.00061	23	3	7	4	0.99801	66	2	11	2+
0.99288	13	4	10	1+	0.99109	15	7	0	1	0.98817	18	5	2	4M
0.98817	18	7	1	1M	0.97580	11	5	8	2	0.97411	12	4	6	4
0.96219	26	6	7	1	0.95928	27	6	6	2	0.95650	9	7	4	0
0.95439	9	1	11	3M	0.95439	9	3	8	4M	0.95345	7	4	1	5
0.95048	12	4	10	2+	0.94902	19	0	0	6	0.94695	9	1	7	5
0.93095	16	4	11	1M	0.93095	16	0	10	4M	0.92746	12	5	5	4M
0.92746	12	5	9	2M	0.92516	16	6	5	3M	0.92516	16	1	13	1M

## Footnotes

1 Certain commercial equipment, instruments, or materials are identified in this paper to foster understanding. Such identification does not imply recommendation or endorsement by the National Institute of Standards and Technology, nor does it imply that the materials or equipment identified are necessarily the best available for the purpose.

## References

[1] Xiao G, Streita FH, Gavrin A, Du YW, Chien CL (1992). Effect of Transition-Metal Elements on the Superconductivity of YBaCuO. Phys Rev B.

[2] Wong-Ng W, Kaiser D, Gale F, Watkins SF, Fronczek F (1990). X-Ray Crystallographic Studies of a Thermomechanically Detwinned Single Crystal of Ba_2_YCu_3_O_6+_*_x_*. Phys Rev B.

[3] Cieplak MZ, Xiao G, Chien CL, Bakhshai A, Artymowicz D, Bryden W, Stalick JK, Rhyne JJ (1990). Incorporation of Gold into YBa_2_Cu_3_O_7_: Structure and *T*_c_ Enhancement. Phys Rev B.

[4] Miceli PF, Tarascon JM, Greene LH, Barboux P, Rotella FJ, Jorgensen JD (1988). Role of Bond Lengths in the 90-K Superconductor: A Neutron Powder-Diffraction Study of YBa_2_Cu_3−_*_x_* Co*_x_* O_7−_*_y_*. Phys Rev B.

[5] Michel C, Er-Rakho L, Raveau B (1981). Les Oxydes La_4−2_*_x_* Ba_2+2_*_x_* Cu_2−_*_x_* O_10−2_*_x_*: Une Structure Inedite Constituee de Groupements CuO_4_ Carres Plans Isoles. J Solid State Chem.

[6] Michel C, Er-Rakho L, Raveau B (1982). Une Nouvelle Famille Structurale: les Oxydes Ln_4−2_*_x_* Ba_2+2_*_x_* Zn_2−_*_x_* O_10−2_*_x_* (Ln = La, Nd). J Solid State Chem.

[7] Michel C, Raveau B (1982). Les Oxydes A_2_BaCuO_5_ (A = Y, Sm, Eu, Gd, Dy, Ho, Er, Yb). J Solid State Chem.

[8] Michel C, Raveau B (1983). Ln_2_BaZnO_5_ and Ln_2_BaZn_1−_*_x_* Cu*_x_* O_5_: A Series of Zinc Oxides with Zinc in a Pyramidal Coordination. J Solid State Chem.

[9] Wong-Ng W, Toby BH, Greenwood W (1998). Crystallization Studies of BaR_2_ZnO_5_ (R = La, Nd, Ho, Er, and Y). Powd Diffr.

[b10-j42kad] 10Powder Diffraction File (PDF), produced by International Centre for Diffraction Data (ICDD), Newtown Square, 12 Campus Blvd., Newtown Square, PA 19073–3273.

[11] Rietveld HM (1969). A Profile Refinement Method for Nuclear and Magnetic Structures. J Appl Cryst.

[b12-j42kad] 12A.C. Larson and R.B. von Dreele, GSAS The General Structure Analysis System, US Government contract (W-7405-ENG-36) by the Los Alamos National Laboratory, which is operated by the University of California for the U.S. Department of Energy, VMS version.

[b13-j42kad] 13Inorganic Structure Data Base (ICSD), published by Fachinformationszentrum Karlsruhe (1994); Collection Code 406331.

[14] Sfreddo CR, Mueller-Buschbaum H (1997). Zur Kristallchemie von Oxozinkat-Platinaten und Oxozinkaten der Zusammensetzung Ba_17_Tm_16_Zn_8_Pt_4_O_37_ and Ba_5_Zn_4_Tm_8_O_21_. Zeit Anorg Allg Chem.

[15] Shannon RD, Prewitt CT (1969). Effective Ionic Radii in Oxides and Fluorides. Acta Cryst.

[16] Shannon RD (1976). Revised Effective Ionic Radii and Systematic Studies of Interatomic Distances in Halides and Chalcogenides. Acta Cryst A.

[17] Michel C, Raveau B (1984). Copper Doped Zinc Oxide Y_2_BaZnO_5_: ESR and Optical Investigation. Mat Res Bull.

[18] Taibi M, Aride J, Darriet J, Moqine A, Boukhari A (1990). Structure Cristalline de l’oxyde Nd_2_BaZnO_5_. J Solid State Chem.

[19] Stalick JK, Wong-Ng W (1990). Neutron Diffraction Study of the ‘Brown Phase’ BaNd_2_CuO_5_. Materials Letters.

[20] Brown ID, Altermatt D (1995). Bond-Valence Parameters Obtained from a Systematic Analysis of the Inorganic Crystal Structure Database. Acta Cryst B.

[21] Brese NE, O’Keeffe M (1991). Bond-Valence Parameters for Solids. Acta Crystallogr B.

[22] Hazen RM, Finger LW, Angel RA, Prewitt CT, Ross NL, Mao HK, Hadidiacos CG (1987). Crystallographic Description of Phases in the Y-Ba-Cu-O Superconductor. Phys Rev B.

[23] Watkins SF, Fronczek FR, Wheelock KS, Goodrich RG, Hamilton WO, Johnson WW (1988). The Crystal Structure of Y_2_BaCuO_5_. Acta Cryst C.

[24] Wong-Ng W, Kuchinskin M, Paretzkin B, McMurdie HF (1989). Crystal Chemistry and Phase Equilibrium Studies of BaO-1/2R_2_O_3_-CuO. I. X-Ray Powder Characterization of BaR_2_CuO_5_ and Related Compounds. Powd Diff.

